# Spliceator: multi-species splice site prediction using convolutional neural networks

**DOI:** 10.1186/s12859-021-04471-3

**Published:** 2021-11-23

**Authors:** Nicolas Scalzitti, Arnaud Kress, Romain Orhand, Thomas Weber, Luc Moulinier, Anne Jeannin-Girardon, Pierre Collet, Olivier Poch, Julie D. Thompson

**Affiliations:** 1 Complex Systems and Translational Bioinformatics (CSTB), ICube Laboratory, UMR7357, University of Strasbourg, 1 rue Eugène Boeckel, 67000 Strasbourg, France; 2 BiGEst-ICube Platform, ICube Laboratory, UMR7357, 1 rue Eugène Boeckel, 67000 Strasbourg, France

**Keywords:** Splice site prediction, Genome annotation, Data quality, Deep learning, Convolutional neural network

## Abstract

**Background:**

Ab initio prediction of splice sites is an essential step in eukaryotic genome annotation. Recent predictors have exploited Deep Learning algorithms and reliable gene structures from model organisms. However, Deep Learning methods for non-model organisms are lacking.

**Results:**

We developed Spliceator to predict splice sites in a wide range of species, including model and non-model organisms. Spliceator uses a convolutional neural network and is trained on carefully validated data from over 100 organisms. We show that Spliceator achieves consistently high accuracy (89–92%) compared to existing methods on independent benchmarks from human, fish, fly, worm, plant and protist organisms.

**Conclusions:**

Spliceator is a new Deep Learning method trained on high-quality data, which can be used to predict splice sites in diverse organisms, ranging from human to protists, with consistently high accuracy.

**Supplementary Information:**

The online version contains supplementary material available at 10.1186/s12859-021-04471-3.

## Background

**Fig. 1:**
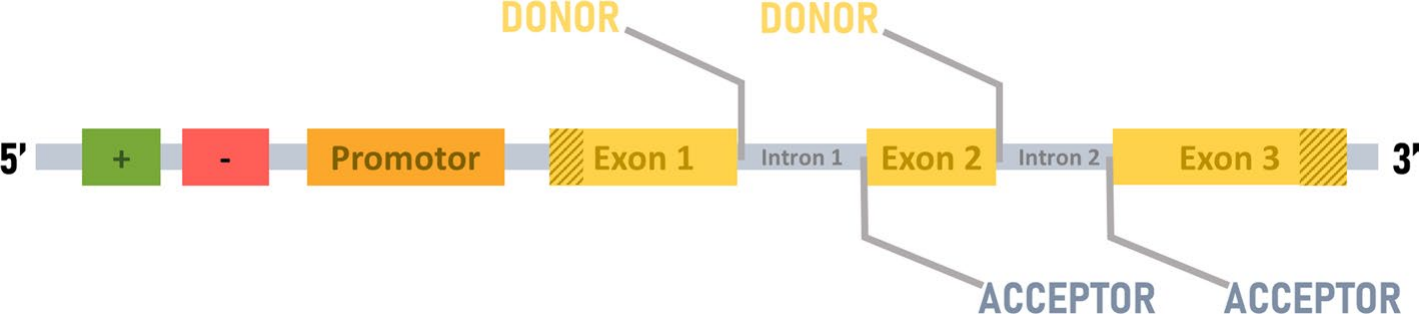
Typical architecture of a eukaryotic protein-coding gene. Green (enhancer) and red (silencer) boxes represent the regulatory elements. The mosaic of exons (labelled yellow boxes) and introns (labelled grey boxes) is usually preceded by a promotor (orange box). The brown diagonal stripes represent the untranslated regions (UTR). The boundaries between exons and introns are called donor splice sites and between introns and exons are acceptor splice sites

The raw genomic sequences generated by next generation sequencing (NGS) are an important source of data for studying and understanding organisms and biological mechanisms. However, without a crucial step of extracting biological knowledge from the raw data, a process called ‘genome annotation’, the sequences are difficult to exploit and may even be useless. A critical step in the annotation process involves the location of genes (i.e. ‘structural annotation’), in particular the protein-coding genes and the characterization of their intron/exon structures. A large number of automatic annotation pipelines have been developed to identify protein-coding genes, such as Braker2 [1], Maker [2] or PASA [3], as well as dedicated resources, such as Ensembl [4] or NCBI [5]. Automatic annotation methods are generally based on a combination of empirical evidence, e.g. mRNA sequencing (RNA-seq) data or known gene structures from closely related organisms, and ab initio gene prediction programs, such as Augustus [6], Genscan [7], Snap [8] or GlimmerHMM [9]. Despite these developments, the annotation of gene structure remains a major challenge, especially for eukaryotic organisms [10–13] due to their complex exon–intron mosaics [14] (Fig. 1).

Eukaryotic gene prediction involves determining the internal architecture of each gene, including the start and stop codons, and the boundaries between each exon and intron, called splice sites (SS). The SS are specifically recognized by the spliceosome, a ribonucleoprotein complex [15], and play an important role in the diversity of the proteome [16, 17]. There are two types of SS, the 5' site (also called donor site) and the 3' site (or acceptor site), located respectively at the exon–intron and intron–exon junctions. SS are generally characterized by the presence of the dinucleotide GT at the 5' site and AG at the 3' site, called canonical sites [18]. The dinucleotides are embedded in longer, consensus motifs: aG**GT**AAGT (Donor) and (Y)6N(C/t)**AG**(g/a)t (Acceptor) [19]. Although the canonical SS are highly conserved [20] and represent more than 98.3% of SS in animals, 98.7% in fungi and 97.9% in plants [21], there are some exceptions, such as the presence of the dinucleotides AT-AC or GC-AG [22, 23], described as non-canonical sites. Thus, the challenges in accurately predicting all SS in a genome are twofold. First, the huge number of GT and AG dinucleotides that are not located at SS can generate a high rate of false positives. Second, the presence of non-canonical SS can lead to false negative predictions if they are not taken into account [24].

A number of methods have been developed to identify SS by exploiting recent high-throughput RNA-seq data, for instance MapSplice [25], TopHat [26] or SplitSeek [27]. However, this approach depends on the availability of high quality data and a minimal depth of sequencing to be able to detect all SS, in particular those in low-expressed isoforms [28]. As a consequence, alternative approaches are needed to identify SS based solely on the genome sequence. Most of them exploit machine learning (ML) algorithms and use several features to describe SS, covering the consensus motifs or other nucleotides in proximity to the SS [29]. The most widely used ML algorithms include Support Vector Machines [30–32], Markov models [33, 34], Random Forest [35, 36] and Bayesian networks [37]. However, these methods are limited by the lack of knowledge about the input sequence (patterns, secondary structures, etc.), complex biological processes [38], a weak genomic context (the region around the SS) and the construction and selection of pertinent feature sets [29], which is often time-consuming. More recently, programs using deep learning (DL) algorithms have been introduced, such as DSSP [39], SpliceRover [40], SpliceFinder [41], or SpliceAI [42]. The DL approaches are based on convolutional neural networks (CNN) and do not require the manual definition of a feature set, because they automatically extract the most pertinent characteristics to classify elements (here, splice sites) in different classes determined by the initial problem [43]. Another advantage of these algorithms is that they are able to find correlations between features in a larger region (i.e. in the genomic sequence). In the context of SS detection, this characteristic is important, as several elements are involved, such as the branchpoint site (BPS), intronic splicing enhancers (ISE), intronic splicing silencers (ISS), exonic splicing enhancers (ESE) and exonic splicing silencers (ESS). Moreover, CNN use fewer parameters than classical multi-layer perceptrons, reducing the risk of overfitting [44], and they also share these parameters to extract local features.

DL methods rely on the availability of high quality data that is pertinent to the problem being solved, in order to train accurate models. For this reason, most of the current SS predictors have been trained on data restricted to humans or other model organisms. To our knowledge, there are no SS prediction tools trained on data from a large range of less well studied organisms, such as insects (except fruit fly), fungi or protists.

In this context, we have developed Spliceator, a new tool for ab initio prediction of eukaryotic multi-species splice sites. Spliceator is based on the CNN technology and more importantly, is trained on an original high quality dataset [45] containing genomic sequences from organisms ranging from human to protists. The training dataset has been rigorously established and validated to reduce the number of errors in the input data and avoid introducing bias in the learning process. This dataset allows us to limit the ‘garbage-in, garbage-out’ effect [46], meaning that poor quality data lead to less reliable results. Based on several benchmark experiments, we show that Spliceator achieves overall high accuracy compared to other state-of-the-art programs, including the neural network-based NNSplice [47], MaxEntScan [48] that models SS using the maximum entropy distribution, and two CNN-based methods: DSSP [39] and SpliceFinder [41]. More importantly, Spliceator performance is robust and remains consistently high for sequences from diverse organisms ranging from human to protists.

## Results

### Design of training and test datasets for multi-species SS prediction

Since we employ a supervised learning approach, the careful construction of the positive and negative datasets used for training the CNN models is essential. We designed eight strategies to build different datasets, where each strategy highlights a parameter that can influence the model performance, such as the input sequence length, the data quality, the type of negative sequences (only false positives (FP) or exon, intron and FP sequences) and the dataset composition, i.e. the effect of balanced or unbalanced datasets with different ratios between the number of positive and negative sequences. Each dataset was then split into separate training and test sets in order to build prediction models for donor and acceptor SS using CNN.

The first dataset, called All Sequences (AS), includes sequences from the 1361 ‘Confirmed’ (error-free) gene sequences available in the G3PO+ dataset (see “Methods” section), as well as the 1380 ‘Unconfirmed’ sequences that contain potential gene prediction errors. The AS dataset is designed to represent real-world problems, in the sense that the data is extracted directly from public databases. For the second dataset, called Gold Standard (GS), we exploited only the ‘Confirmed’ sequences implying that this set is error-free. The resulting AS and GS positive subsets include donor and acceptor SS from human, as well as from a diverse set of 147 eukaryotic organisms, ranging from primates to protists. In order to construct a robust negative subset of non-SS sequences, we again exploited both ‘Confirmed’ and ‘Unconfirmed’ sequences for the AS dataset and only ‘Confirmed’ sequences for the GS dataset. To do this, we randomly selected sequences from the exon/intron regions of the G3PO+ genomic sequences, as well as sequences containing GT/AG dinucleotides that are not SS.

In order to verify that there is no over-representation of sequences from specific organisms or clades, we calculated the mean pairwise percent identity for the input sequences with a length of 600 nucleotides (nt) (Table 1) and showed that the majority (> 90%) of the sequences in each positive subset (AS and GS) for both donor and acceptor SS share between 20 and 30% identity. We also calculated the mean pairwise percent identity for sequences with a length of 20 nt, i.e. specifically the short region around SS. Again, the pairwise percent identity is similar for AS and GS positive datasets, however the majority of these sequences share between 20 and 60%, showing that the context close to the SS is more conserved. Interestingly, the donor sequences of length 20 nt are more conserved than acceptor sequences, e.g. in the GS dataset, 37.17% of donor sequences share 40–50% mean identity compared to 34.09% of acceptor sequences.

**Table 1:** Distribution of sequences according to the mean percent identity

Pairwise sequence identity	Sequence length: 600 nt				Sequence length: 20 nt			
	Donor		Acceptor		Donor		Acceptor	
	AS	GS	AS	GS	AS	GS	AS	GS
0–10%	0.0%	0.0%	0.0%	0.0%	0.04%	0.03%	0.04%	0.03%
10–20%	0.35%	0.27%	0.33%	0.25%	1.0%	0.71%	1.65%	1.32%
20–30%	**92.77%**	**94.88%**	**92.45%**	**94.7%**	10.27%	8.93%	13.92%	12.44%
30–40%	6.83%	4.78%	7.18%	4.99%	32.62%	31.57%	**34.05%**	33.20%
40–50%	0.03%	0.04%	0.03%	0.04%	**36.24%**	**37.17%**	32.89%	**34.09%**
50–60%	0.01%	0.01%	0.01%	0.01%	16.28%	17.47%	14.22%	15.34%
60–70%	0.0%	0.0%	0.0%	0.0%	3.2%	3.65%	2.9%	3.2%
70–80%	0.0%	0.0%	0.0%	0.0%	0.3%	0.39%	0.29%	0.33%
80–90%	0.0%	0.0%	0.0%	0.0%	0.03%	0.04%	0.03%	0.03%
90–100%	0.0%	0.0%	0.0%	0.0%	0.02%	0.03%	0.02%	0.02%

Pairwise sequence percent identity of positive subsets (AS: All Sequences and GS: Gold Standard) for sequences with a length of 600 nt and 20 nt for donor and acceptor SS (values in bold correspond to the highest percentage of identity)

### Impact of genomic context

**Fig. 2:**
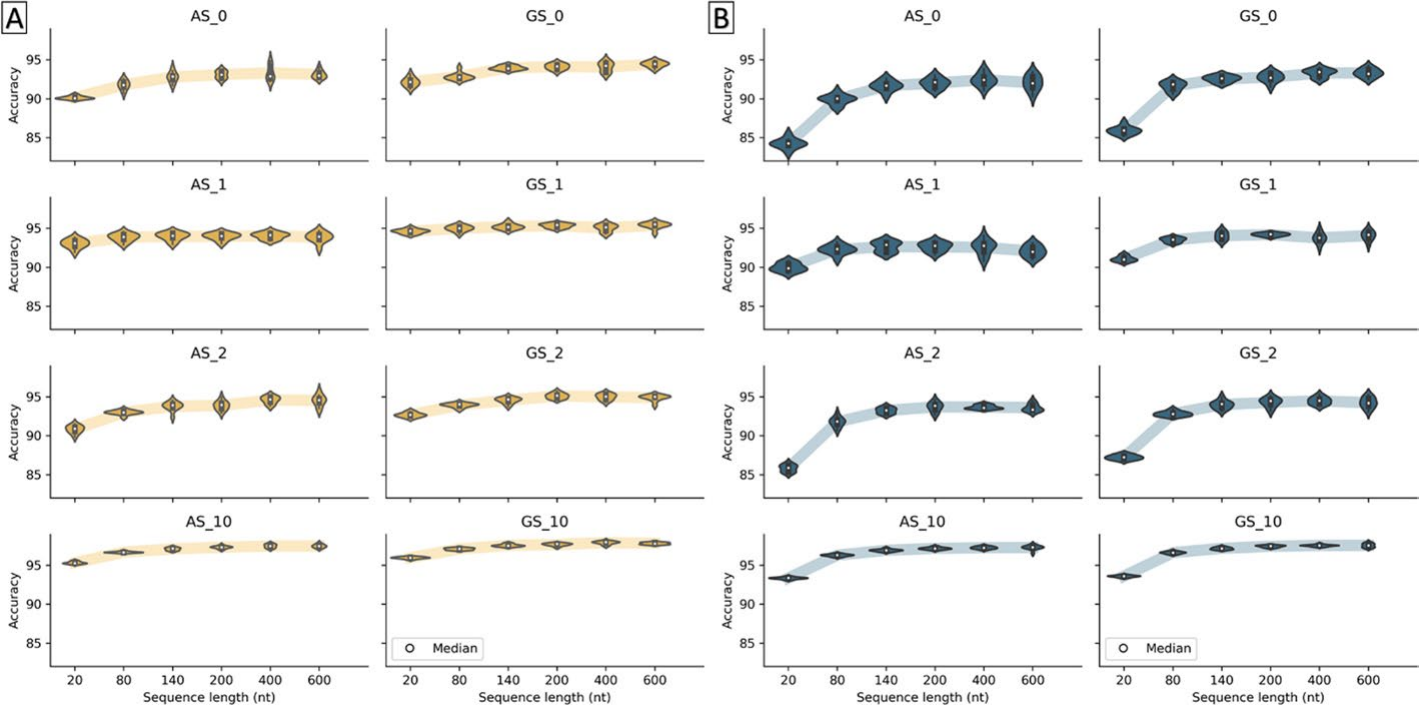
Prediction accuracy according to input sequence length for each dataset (AS: All Sequences and GS: Gold Standard) for **A** donor and **B** acceptor SS

To evaluate the impact of the genomic context around the SS on the prediction performance of our CNN method, we constructed subsets of sequences for the AS and GS datasets having different lengths, ranging from 20 to 600 nt. The sequence segments upstream and downstream of the SS dinucleotide contain information allowing the discrimination of SS and non-SS, such as the BPS, polypyrimidine tract (PPT) or regulatory *cis*-elements including exon/intron splicing enhancers or silencers (ESE/ISE or ESS/ISS) [49]. Determining a pertinent sequence length is important because too short genomic regions would prevent the model from using important discriminatory sites, while too large genomic regions may introduce noise-inducing features and loss of accuracy [50]. We then built CNN prediction models for donor and acceptor SS, using these different sequence lengths. Figure 2 (and Additional file 2: Table S1 and S2) summarizes the prediction accuracies obtained by the models for each SS on the test sets.

We observe similar trends for the prediction of donor and acceptor SS with the AS and GS datasets. The average prediction accuracy increases for sequence lengths ranging from 20 to 200 nt and then generally levels off, indicating that the model cannot find relevant genomic context features beyond this length. However, there are some differences between the datasets with different compositions (described in detail below). For example, for the AS_10 and GS_10 datasets (ratio 1:10 of positive to negative examples), the prediction accuracies are more homogeneous and higher than the other datasets. Interestingly, the sequence length has less effect for the AS_1 and GS_1 datasets, compared to AS_0 and GS_0 respectively. AS_1 (respectively GS_1) has the same balanced ratio of positive to negative examples as AS_0 (respectively GS_0), but the negative examples are more heterogeneous, consisting of exon, intron and FP sequences.

Based on this initial analysis, in the following experiments, we used a sequence length of 200 nt for the prediction of donor and acceptor sites with the AS and GS dataset, to consider a genomic context that is neither too small nor too large.

### Impact of data quality

**Fig. 3:**
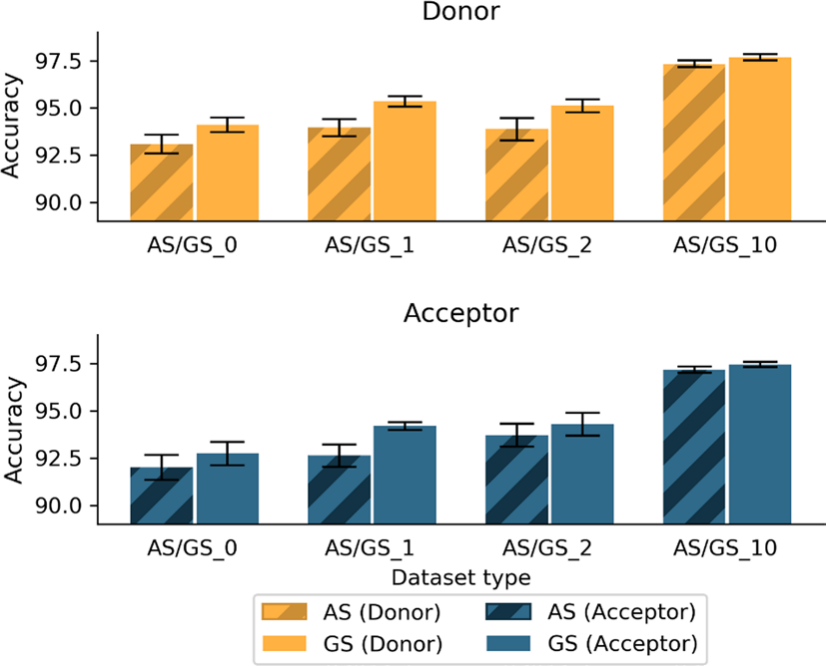
Average prediction accuracy for donor and acceptor SS, using the AS and GS datasets (AS/GS_0 = positive/negative ratio of 1:1 with only FP sequences in negative subset; AS/GS_1 = positive/negative ratio of 1:1 with exon, intron and FP sequences; AS/GS_2 = positive/negative ratio of 1:2 with only FP sequences in negative subset; AS/GS_10 = positive/negative ratio of 1:10 with only FP sequences in negative subset). Standard deviations are indicated by black bars

As described above, the GS dataset contains only true SS from the ‘Confirmed’ gene sequences, while the AS dataset includes some noise (i.e. false SS) from ‘Unconfirmed’ sequences. To estimate the impact of this noise on model prediction, we compared the average accuracy of the AS models with the corresponding GS models for each SS (donor and acceptor) and for different dataset compositions, as shown in Fig. 3 (Additional file 1: Table S1).

As expected, the GS models achieved the best accuracy with an average of 94.11%, 95.34%, 95.11% and 97.68% respectively for the GS_0, GS_1, GS_2 and GS_10 donor datasets and 92.73%, 94.19%, 94.59%, 97.45% respectively for the acceptor datasets. In comparison, the AS models obtained an average accuracy of 93.09% (-1.02%), 93.95% (-1.39%), 93.88% (-1.23%) and 97.33% (-0.35%) respectively for the AS_0, AS_1, AS_2 and AS_10 donor datasets, and 91.99% (-0.74%), 92.62% (-1.57%), 93.70% (-0.49%) and 97.17% (-0.28%) for the acceptor datasets. The results of unpaired t-tests (Additional file 1: Table S2) show that all differences between AS and GS datasets are statistically significant. Interestingly, the difference between AS_1 and GS_1 is the largest for both donor and acceptor models. Based on these results, we selected only the GS models for the following experiments.

### Impact of negative dataset composition

**Fig. 4:**
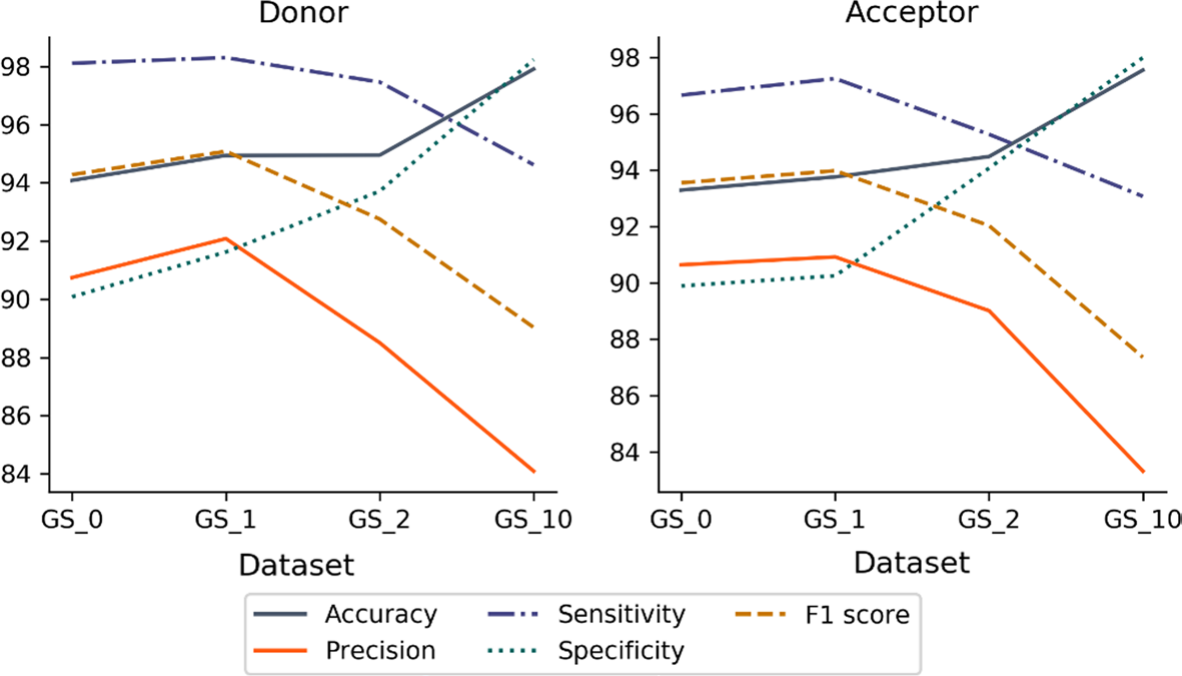
Average values of the 5 performance metrics (accuracy, precision, sensitivity, specificity and F1 score) for each dataset composition and for each type of SS (donor or acceptor). GS_0 = positive/negative ratio of 1:1 with only FP sequences in negative subset, GS_1 = positive/negative ratio of 1:1 with exon, intron and FP sequences in negative subset, GS_2 = positive/negative ratio of 1:2 and GS_10 = positive/negative ratio of 1:10

While the definition of reliable positive examples is clearly essential, the construction of the negative dataset will also have an impact on the ability of CNN methods to distinguish between positive and negative examples. The prediction of SS is an intrinsically unbalanced problem, since in a protein coding gene the SS represent only a small proportion of the total nucleotide length. Therefore, to investigate the impact of the negative subset on prediction performance, we constructed a number of datasets with different types of negative sequences and different ratios of positive and negative examples. We designed two balanced datasets, both with a ratio 1:1 of positive to negative examples, but with either homogeneous (GS_0) or heterogeneous (GS_1) negative examples, as well as two unbalanced datasets with ratios of 1:2 (GS_2) and 1:10 (GS_10) of positive to negative examples. The unbalanced datasets both have heterogeneous negative examples. We then computed different metrics to evaluate the performance of each model on the test set, as shown in Fig. 4.

The overall best performance for the test set is obtained using the GS_1 dataset, with a balanced number of positive and negative sequences and heterogeneous negative examples (exon, intron and FP sequences). Although the average accuracy is lower than for the unbalanced GS_10 dataset (positive/negative ratio of 1:10), the other metrics including the average F1 score and precision are higher for the GS_1 balanced dataset.

To confirm these results, the GS models were also evaluated on a set of 5 independent benchmarks (human, fish, fly, worm, plant) and using different metrics. The results are shown in Additional file 1: Figure S1A and B. The GS_1 dataset again obtains better overall performance metrics for both donor and acceptor SS, notably for fly, worm and plant species. Based on these results, we chose to consider only the GS_1 models in the following experiments.

### Performance of optimized CNN model

**Fig. 5:**
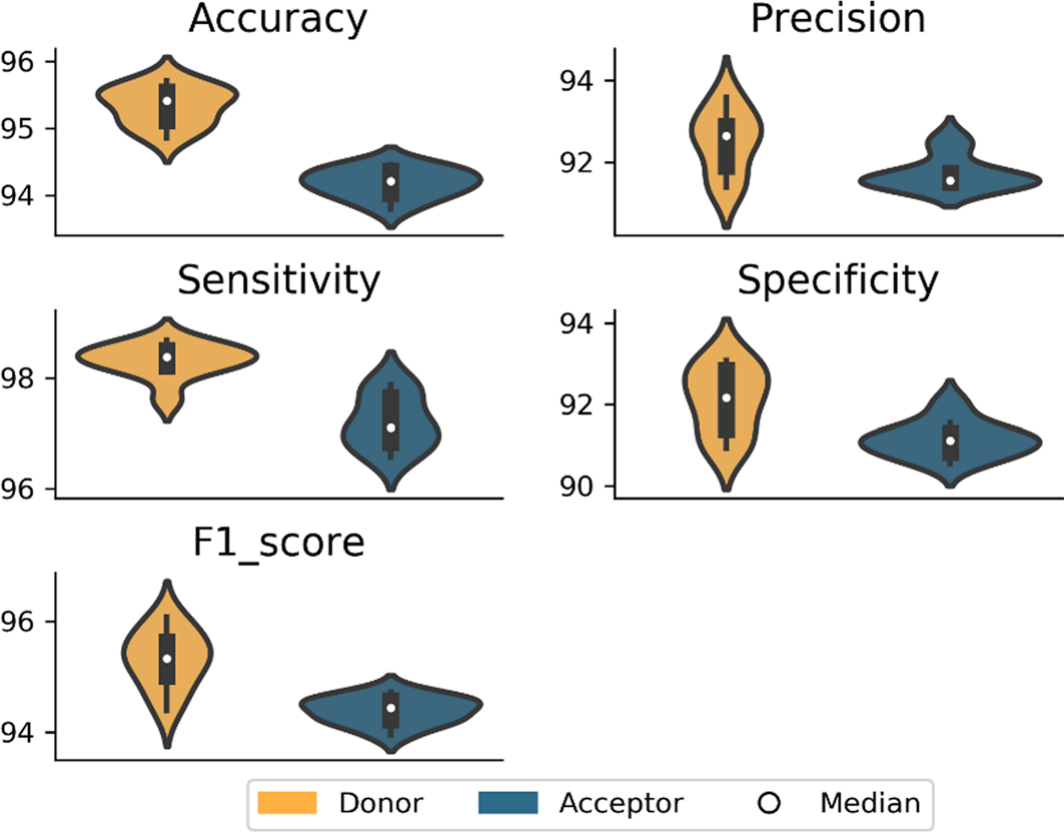
Performance of optimized model (GS_1 dataset, positive/negative ratio of 1:1 with heterogeneous negative examples and input sequence length = 200 nt) averaged over 10 experiments

Based on our initial analyses, we determined the optimal training set for the CNN models to predict donor and acceptor SS, namely the GS_1 dataset: a high quality balanced dataset with an equal number of positive and negative sequences, heterogeneous negative examples containing exon, intron and FP sequences and an input sequence length of 200 nt. For this optimized model, we further characterized the prediction performance of Spliceator averaged over a total of 10 experiments due to the random selection of negative sequences. The results are shown in Fig. 5 (Additional file 1: Table S3).

The average accuracy is 95.34% for the optimized donor model and 94.19% for the acceptor model. The precision of the models is similar, ranging from 92.50% (donor) to 91.73% (acceptor). We observed high sensitivity for both models, with 98.31% (donor) and 97.20% (acceptor), although the specificity is slightly lower, with 92.11% (donor) and 91.14% (acceptor). Finally, the F1 scores are similar for both donor and acceptor with 95.32% for donor and 94.39% for acceptor SS. Thus, the average performance for the donor model is slightly higher than for the acceptor model, which might be explained by the fact that the donor SS consensus motif is more conserved than the acceptor SS motif (Table 1). The acceptor SS also contains a low complexity PPT sequence, which may complicate predictions.

### Model interpretability

**Fig. 6:**
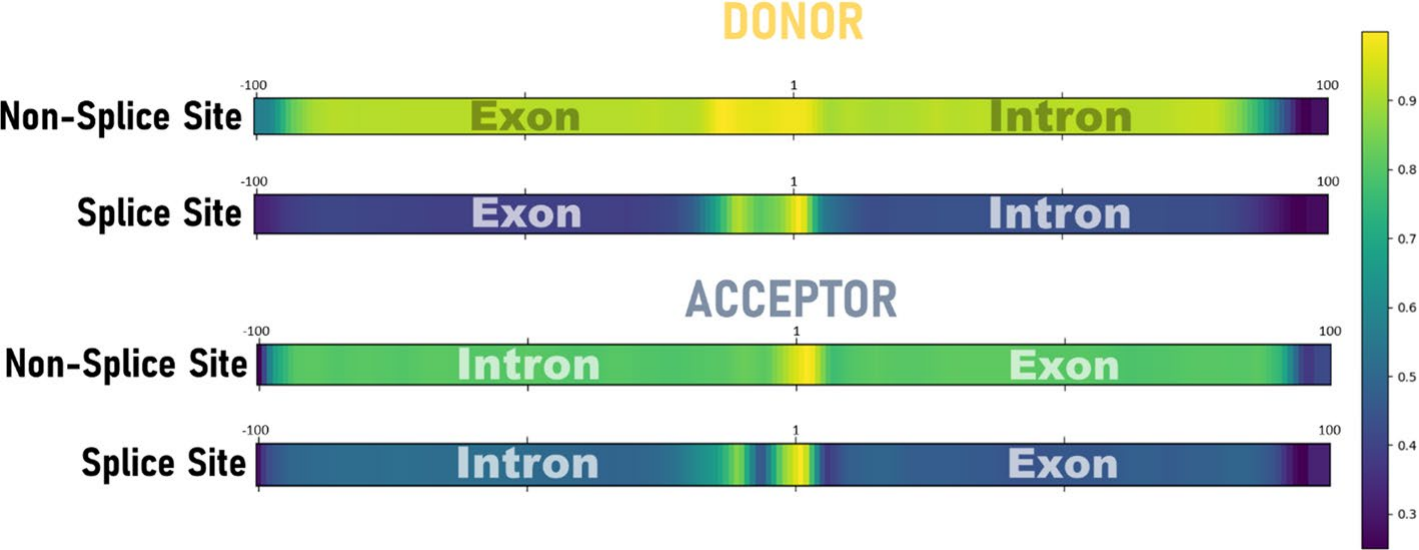
Average heatmap of the two classes, non-Splice Site and Splice Site, for donor and acceptor SS, with colors ranging from yellow (very important nucleotide position) to dark blue (not important position). The dinucleotide characterizing the SS is located at positions 101–102 for the donor and acceptor SS

In this section, we focus on the nucleotide regions that influenced the models during the learning step. Using the Grad-CAM method (see “Methods” section), we measured the impact of each nucleotide position in the input sequences, thus allowing us to highlight the most important regions of these sequences that are determining factors in the learning step of the model. We calculated 10,000 heatmaps per class and the average heatmap for each SS model (donor and acceptor) and each class (non-SS, SS) is shown in Fig. 6. Therefore, this representation shows only the most important features that the models use.

The heatmaps show that in order to classify sequences as non-SS, both models are based on elements of the whole sequence (score > 0.8) with the exception of the 5' and 3' extremities (score < 0.4, probably due to the CNN processing), although we observe a higher score (> 0.9) close to the positions 1–2. The heatmaps for sequences containing donor or acceptor SS are more specific than for the non-SS sequences. For the donor sequences, the region around the SS (~ 10 nt upstream and downstream) seems to be more influential, with a predominance for the upstream exonic side. The positions 1 and 2, representing the GT dinucleotide, have the highest scores as expected. For the acceptor sequences, the central region around the AG dinucleotide is also the most important, although it is less well delineated than for donor SS. We observe an upstream intronic region of about 10 nt that seems to slightly impact learning (score > 0.6), which probably corresponds to the PPT. A second upstream region from position -50 is also influential, possibly covering the BPS known to be generally located around 40 nt upstream of the acceptor site [51], although some BPS may be more distant up to a distance of 400 nt [52, 53]. Interestingly, the downstream exonic region also seems to play a role in the training process for the acceptor SS (score > 0.5).

### Comparison with existing SS prediction methods

In order to compare the performance of the Spliceator models with other state-of-the-art methods, namely NNSplice, MaxEntScan, DSSP and SpliceFinder, we used six independent benchmarks from a wide range of organisms (see “Methods” section). The performance metrics are shown in Table 2.

**Table 2:** Performance of Spliceator and state-of-the-art programs on six independent benchmarks

	Accuracy		Precision		Sensitivity		Specificity		F1 Score	
	Donor	Acceptor	Donor	Acceptor	Donor	Acceptor	Donor	Acceptor	Donor	Acceptor
Human										
Spliceator	92.7	89.5	90.0	86.1	96.1	94.4	89.4	84.7	**93.0**	90.0
SpliceFinder	91.5	**91.1**	76.9	73.3	**97.0**	**99.8**	89.6	88.2	85.8	84.5
DSSP	**93.2**	91.0	**96.9**	**94.4**	89.2	87.1	**97.2**	**94.8**	92.9	**90.6**
MaxEntScan	91.8	83.7	88.8	77.3	95.8	95.6	87.9	71.9	92.1	85.5
NNSplice	68.4	65.9	62.3	61.5	89.2	86.7	48.5	44.8	73.4	72.0
Fish										
Spliceator	**95.0**	91.3	91.6	86.0	**99.0**	98.7	90.9	84.0	**95.1**	91.9
SpliceFinder	92.8	**94.9**	81.5	85.0	96.4	**99.8**	91.4	93.0	88.3	91.8
DSSP	94.6	93.6	**97.5**	**94.2**	91.5	92.9	**97.6**	**94.3**	94.4	**93.6**
MaxEntScan	93.9	83.3	90.6	75.4	98.1	99.0	89.8	67.7	94.2	85.6
NNSplice	71.0	68.9	64.3	63.9	90.8	85.6	52.2	52.5	75.3	73.2
Fly										
Spliceator	**94.6**	90.4	91.6	86.2	**98.3**	96.4	91.0	84.5	**94.8**	91.0
SpliceFinder	92.1	90.9	79.8	72.8	95.8	**99.8**	90.7	88.0	87.1	84.2
DSSP	94.0	**91.6**	**96.6**	**93.1**	91.3	89.8	**96.7**	**93.4**	93.9	**91.4**
MaxEntScan	94.5	86.0	91.8	79.5	97.6	97.0	91.3	75.0	94.6	87.4
NNSplice	71.7	71.1	65.3	64.8	91.4	92.1	52.3	50.3	76.2	76.1
Worm										
Spliceator	**93.9**	**88.8**	91.4	86.9	**96.9**	91.4	90.9	86.2	**94.1**	**89.1**
SpliceFinder	88.7	87.9	71.2	64.1	93.3	**99.3**	87.1	84.8	80.8	77.9
DSSP	90.4	84.1	**97.0**	**93.1**	83.4	73.6	**97.4**	**94.5**	89.7	82.2
MaxEntScan	92.9	82.0	91.6	76.8	94.5	92.0	91.3	72.1	93.0	83.7
NNSplice	65.8	57.8	61.9	58.0	80.3	56.4	51.7	59.2	69.9	57.1
Plant										
Spliceator	**94.7**	**90.6**	91.8	89.1	**98.1**	92.7	91.3	88.6	**94.9**	**90.8**
SpliceFinder	88.9	87.6	72.2	63.1	92.9	**99.3**	87.5	84.4	81.3	77.2
DSSP	88.2	85.1	**96.5**	**94.2**	79.3	74.8	**97.1**	**95.4**	87.1	83.3
MaxEntScan	92.4	85.3	90.5	79.2	94.6	95.9	90.1	74.8	92.5	86.7
NNSplice	59.8	59.2	57.2	57.1	78.4	71.3	41.0	47.3	66.2	63.4
PVP										
Spliceator	86.0	83.5	82.9	83.1	**90.8**	84.0	81.2	82.9	**86.6**	**83.6**
SpliceFinder	**87.3**	**89.3**	67.9	58.8	77.6	**98.6**	89.9	87.7	72.4	73.8
DSSP	84.3	76.6	**92.5**	**84.7**	74.6	65.0	**93.9**	**88.2**	82.6	73.5
MaxEntScan	84.5	80.0	81.0	75.4	90.1	89.4	79.1	70.4	85.2	81.8
NNSplice	64.8	63.7	60.4	61.4	85.8	73.9	43.6	53.5	70.9	67.1
Average										
Spliceator	**92.82**	89.02	89.88	86.23	**96.53**	92.93	89.12	85.15	**93.08**	**89.40**
SpliceFinder	90.22	**90.28**	74.92	69.52	92.17	**99.43**	89.37	87.68	82.62	81.57
DSSP	90.78	87.00	**96.17**	**92.28**	84.88	80.51	**96.65**	**93.43**	90.10	85.77
MaxEntScan	91.67	83.38	89.05	77.27	95.12	94.82	88.25	71.98	91.93	85.12
NNSplice	66.92	64.43	61.90	61.12	85.98	77.67	48.22	51.27	71.98	68.15

Performance metrics for Spliceator, SpliceFinder, DSSP, MaxEntScan, and NNSplice, using six independent benchmarks from model and non-model organisms: human, fish, fly, worm, plant and PVP (Protists + Viridiplantae) (values in bold correspond to the highest performance)

**Fig. 7:**
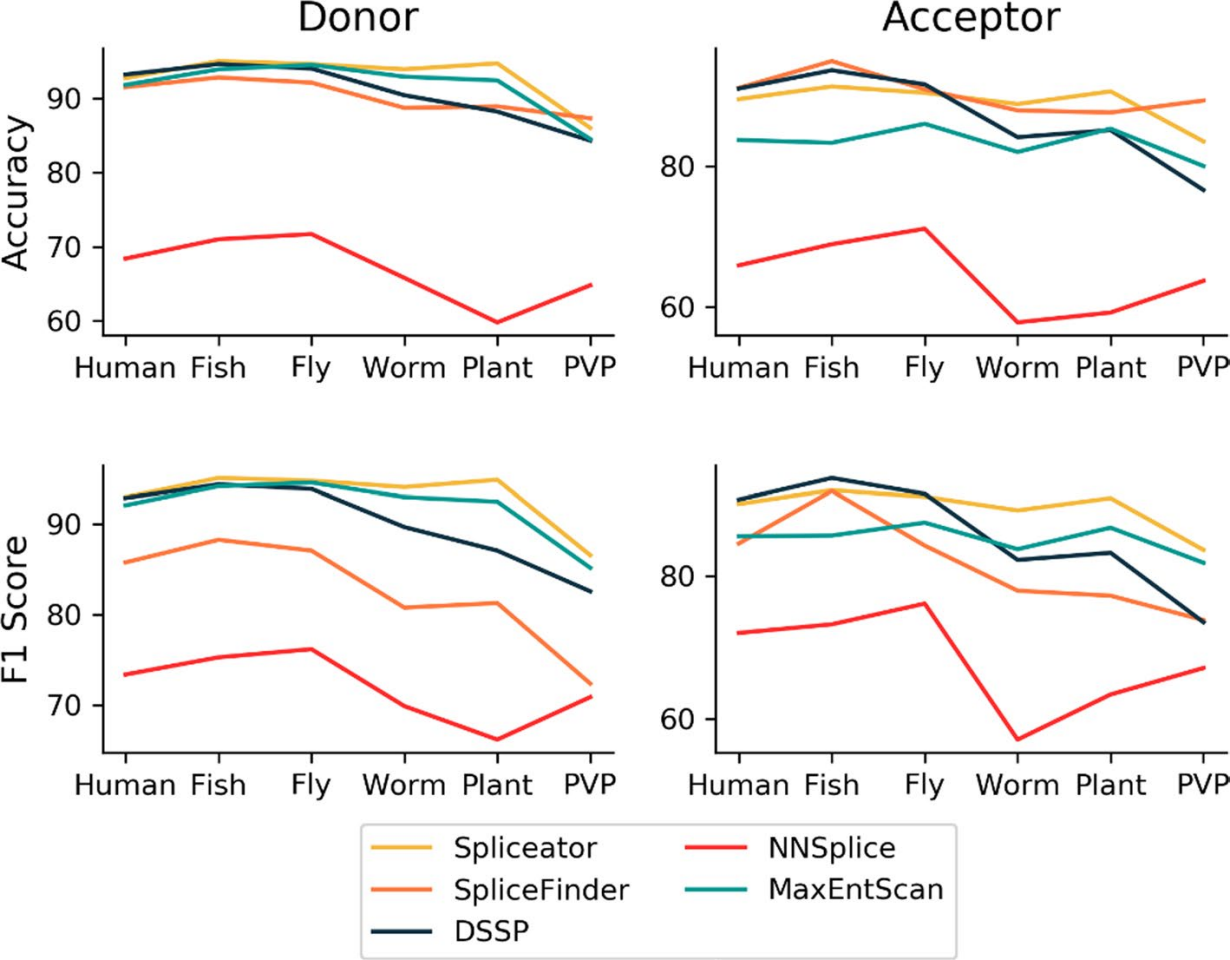
Accuracy and F1 score for each program and for each independent benchmark representing diverse organisms

For the donor SS prediction, Spliceator obtains the best average accuracy of 92.82%, with an average increase of + 25.9, + 2.6, + 2.04 and + 1.15% compared to NNSplice, SpliceFinder, DSSP and MaxEntScan respectively. For the acceptor SS prediction, Spliceator obtains the second best average accuracy, with 89.02% (− 1.26%) compared to SpliceFinder with 90.28%, although Spliceator is more accurate on the Worm and Plant benchmarks. To further investigate the reasons for the different performances, we considered four other metrics, including the precision, sensitivity, specificity and F1 score. A high precision indicates that the program predicts few FP. Spliceator obtains the second best average precision of 89.88% for the donor SS and 86.23% for the acceptor SS, behind DSSP (96.17% for donor and 92.28% for acceptor SS). SpliceFinder, which has generally good accuracy, obtains lower precision (74.92% for the SS donor and 69.52% for the SS acceptor). Sensitivity and specificity are two inseparable metrics. They describe the proportion of well predicted elements and their quality, i.e. if elements have been correctly predicted.. The F1 score combines the precision and the sensitivity metrics and provides a more global view of the number of correctly predicted elements. Spliceator obtains the best average F1 score for both donor and acceptor SS, with 93.08% (+ 1.15%) and 89.4% (+ 3.83%) compared to the second best program for donor SS, MaxEntScan with an F1 score of 91.93% or acceptor SS, DSSP with a F1 score of 85.57%. Figure 7 shows the accuracy and F1 score for each program on the individual benchmarks containing SS from different organisms. While most of the programs tested achieve high scores on the vertebrate sequences (human and fish), reflecting their training sets focused on human SS, Spliceator performance is generally better on the more distant organisms (worm, plant and PVP).

Finally, for each model, we observe that the predictions of the donor SS are slightly better than those of the acceptor SS. This difference is probably due to the divergence of the acceptor SS motif, but also to the genomic context around it which seems to be more complex.

## Discussion

Thanks to high-throughput technologies, as well as the development of computing power, huge amounts of data can now be exploited by DL algorithms and produce remarkable results [54–57]. In particular, CNN are increasingly used in the field of bioinformatics [58–60], for example to detect specific patterns in a genomic sequence [61] where the reduced number of parameters allows for better generalization compared to other ML methods. Moreover, maxpooling techniques allows the algorithm to focus on the local features that it considers most important.

In this context, we have developed a SS prediction program called Spliceator, based on a three-layer convolutional CNN. Despite the recent use of RNA-seq to accurately identify SS, it is currently impossible to obtain experimental data for the full panel of transcripts for all tissues and all developmental stages. Thus, ab initio SS prediction programs that rely only on the genomic sequence remain essential. Clearly, it would be ideal to couple ab initio prediction programs such as Spliceator with RNA-seq based programs in genome annotation tools or workflows.

The accuracy of ab initio algorithms is dependent on the quality of the data used during the training step. Indeed, although neural networks are getting deeper and more complex [62, 63], the fact remains that data is the cornerstone of artificial intelligence. Consequently, the majority of SS prediction programs use sequence data from humans (such as GRCh38 or HS3D [64]) or other model organisms [36, 39, 65], where high quality, expert-refined data is available. For non-model organisms, it is more difficult to find accurate training data and very few CNN methods have been designed specifically to predict SS in non-model organisms. Since SS and other regulatory motifs may be conserved across similar species [20], some work has been done to try to transfer models trained on model organisms to related organisms, for example between different vertebrate genomes [66]. Others have built cross-species models for specific clades, such as animals or plants using Helixer [67], but unfortunately the source code for this program is not yet stable (according to the authors). The aim of our work was to extend the idea of cross-species models to conceive universal SS prediction models (one for each SS), that are applicable to a wider range of organisms.

For the training of the Spliceator models, we focused on the construction of a multi-species dataset that is as representative as possible of the eukaryotic domain (from primates to protists). This dataset is based on an extension (G3PO+) of the gene prediction benchmark G3PO. Since high-quality, genome-wide annotations are not available for the 147 species in this dataset, we developed a protocol based on expert-guided comparative sequence analysis in order to identify reliable SS in a subset of genes. Since the G3PO+ gene sequences are evolutionarily related, we eliminated redundant sequences, which could cause potential bias of sequence over-representation and thus a risk of overfitting. We also made an effort to respect the proportions of non-canonical SS (2.2% donor and 1.3% acceptor) found in real-world data [21].

To investigate the impact of the quality of the initial training data on the CNN models, we extracted data from public databases such as Ensembl [4] and UniProt [68], where it has been estimated that many proteins (with the exception of Swiss-Prot, which represents 0.3% of UniProt) have errors [69]. We then built a dataset called ‘All Sequences’ (AS), that includes some badly predicted gene sequences [45] and thus introduces noise in the form of wrong or missing SS. We compared the CNN model trained on the AS dataset with a second model trained on a ‘Gold Standard’ (GS) dataset, which was cleaned by removing all error-prone sequences. Since our results showed that the quality of the data had a significant impact on the accuracy of the models, we conclude that quality control and data cleaning steps are essential in order to obtain better results.

We also tested the impact of other parameters, such as the length of the input sequences. It is important to carefully select the size of the genomic sequence in order to take into account different important elements such as regulatory elements (ESE, ESS, ISE or ISS [70]), the BPS [71] and the PPT [22] that can be kept and help the algorithm to generalize. All these elements constitute intrinsic signals that are indispensable for the spliceosome to accurately recognize the SS. In order to include enough *cis* elements without introducing too much noise, we chose an input sequence length of 200 nt for both donor and acceptor models. Unfortunately, many other external signals impacting SS recognition by the spliceosome cannot be detected by current methods such as the secondary structure of RNA [72], or the transcription speed of polymerase II [73].

Finally, we investigated the impact of the negative examples and the use of balanced or unbalanced datasets, in terms of the ratio of positive to negative examples. SS prediction is an inherently unbalanced problem, because the number of nucleotides involved in a SS is much smaller than the number of non-SS nucleotides. The results confirmed the hypothesis that an unbalanced dataset was more prone to overfitting because one of the classes is overrepresented [66]. Moreover, the heterogeneous negative examples provided better performance. All these tests allowed us to optimize our method and improve prediction performance.

In order to estimate the performance of Spliceator on independent genome data, we used six different benchmarks from diverse organisms and compared Spliceator with a number of state-of-the art programs, including two other CNN-based methods. As expected, the more recent CNN-based methods generally achieved higher performance metrics than the older prediction methods that used either neural networks or maximum entropy distributions approaches. We calculated a number of different performance metrics, since the most suitable metric to measure ‘good’ performance will depend on the specific user application. For example, accuracy is useful when the true positives and true negatives are more important, while the F1 score is used when the false positives and false negatives are crucial. Spliceator achieved the highest accuracy (92.82%) for the donor SS, and the second best accuracy (89%) for the acceptor SS. In terms of F1 score, Spliceator outperformed the current state-of-the art programs for both donor (93.08%) and acceptor (89.40%) SS. Interestingly, Spliceator performed very well on the human benchmark even though it was trained with only 45 human genes. However, a major strength of Spliceator is that it maintains good performance over a wide range of organisms, from human to protists. Our results thus showed that a universal SS prediction program is feasible, and hopefully performance can be further increased in the future by including more divergent species data in the model.

## Conclusions

Here, we present a new approach to train Spliceator, a universal splice site prediction program based on a high-quality dataset from diverse eukaryotic organisms (from primates to protists). We highlighted the inherent link between data quality and the performance of prediction programs based on machine learning algorithms. We also showed that including high quality multi-species data can result in accuracy equivalent to other state-of-the-art SS prediction programs. In the future, it would be interesting to include more data from other species, but also to test other types of network architecture in order to extract new high-level features. Moreover, as some of the extracted features are highly conserved, it would be interesting to use our model to perform transfer learning for gene annotation of other organisms.

## Methods

### Data collection

Initial datasets were constructed for each type of SS, in order to establish two separate models: one to predict donor SS and one to predict acceptor SS. The models developed in this study are based on supervised learning allowing the classification of entries in two classes (0: nucleotide not involved in SS, and 1: nucleotide involved in SS). Thus, each dataset is constructed from a positive subset containing SS sequences and a negative subset containing non-SS sequences.

To build the positive and negative subsets, gene sequences from the multi-species benchmark G3PO [45] were used. G3PO is based on 147 phylogenetically disperse organisms and contains 1793 sequences including 20 human Bardet-Biedl Syndrome (BBS) genes (Additional file 1: Table S4) and their orthologous sequences (ranging from primates to protists) extracted from the OrthoInspector database v3.0 [74]. Following the same methodology implemented in G3PO, we extended the original dataset by adding 948 sequences from 25 human genes responsible for myopathies and their orthologs from 47 metazoans (Additional file 1: Figure S2). The 948 protein sequences in the extended dataset (called G3PO+) were analyzed according to the G3PO protocol in order to classify them into two categories: those without gene prediction errors (called ‘Confirmed’) and those that contain at least one error (called ‘Unconfirmed’). Errors include insertions, deletions and mismatches in the N-terminal, C-terminal or internal regions. This protocol allows to verify the quality of the data and ensures that the SS present in the ‘Confirmed’ sequences are biologically true, i.e. they are recognized by the spliceosome. Table 3 summarizes the composition of the G3PO+ dataset.

**Table 3:** Composition of the original G3PO and extended G3PO+ datasets

	G3PO	Extension	G3PO+
Confirmed	889	472	1361
Unconfirmed	904	476	1380
Total	1793	948	2741

To build the G3PO+ dataset, we retrieved orthologous sequences for 45 human genes and performed multiple sequence alignments. Each sequence was then checked to identify those that contained no errors, called ‘Confirmed’, and those that contained at least one error, called 'Unconfirmed'

### Construction of a series of training and test sets

**Fig. 8:**
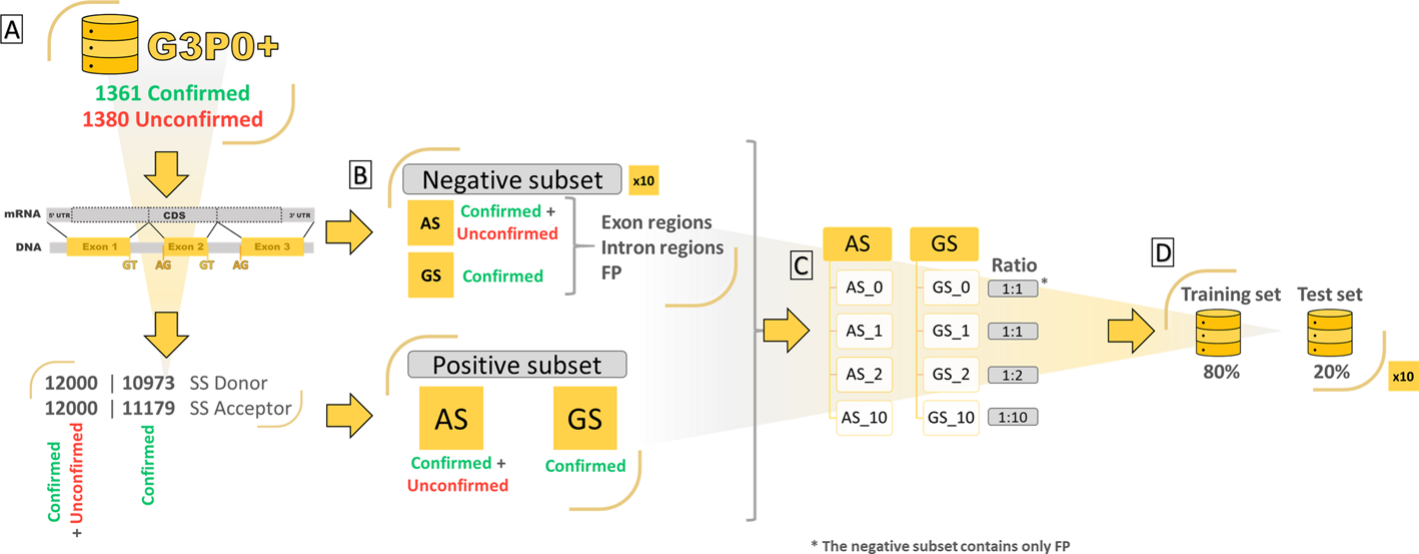
Overview of the construction of the training and test sets. **A** DNA sequences and exon maps are recovered for each G3PO+ gene. **B** The AS (All Sequences) positive subset includes the SS of all G3PO+ ‘Confirmed’ and ‘Unconfirmed’ sequences. The GS (Gold Standard) positive subset includes only the SS of the ‘Confirmed’ sequences. Ten negative AS subsets and ten negative GS subsets are then constructed by random sampling of the exon, intron and FP regions of the corresponding genomic sequences. **C** Four AS and four GS datasets are then constructed with different ratios of positive and negative SS (described in Table 4). **D** Finally, the training and test sets are formed by shuffling the positive and negative sequences (10 times for each AS and GS dataset)

Based on the gene sequences in the G3PO+ benchmark, we constructed a series of datasets used to train the Spliceator models and estimate the effect of various parameters on their prediction performance. Figure 8 shows an overview of the dataset construction process. The positive and negative subsets are described in the following sections.

### Positive subsets

The 2741 G3PO+ sequences are classified into 2 categories, either 'Confirmed' (1361 sequences) because they were annotated 'error-free', or 'Unconfirmed' (1380 sequences) because they contained at least one gene prediction error. For each G3PO+ sequence, genomic sequences and exon maps were retrieved from the Ensembl database [4] release 87, and the SS were extracted, flanked by a ± 300 nt environment. A verification was made to ensure that no sequences containing undetermined nucleotides (noted 'N') were selected. The GS datasets contain only SS from the 1361 'Confirmed' sequences. Thus, the same positive subset for each GS dataset (GS_0, GS_1, GS_2 and GS_10) contains 10,973 donor and 11,179 acceptor SS sequences, where each sequence is of length 600 nt with the GT (donor) or AG (acceptor) dinucleotide in the central position (301 and 302). In contrast, the AS datasets contain SS from all 2741 G3PO+ sequences, including both ‘Confirmed’ and ‘Unconfirmed’ sequences. Thus, the AS datasets are representative of the data present in the public databases, as no pre-processing has been performed on the data, and therefore they include a certain number of errors. To eliminate any bias from the size of the datasets, an equivalent number of SS sequences were used (12,000 donor and 12,000 acceptor) to form the same positive subset for each AS dataset (AS_0, AS_1, AS_2 and AS_10).

**Fig. 9:**
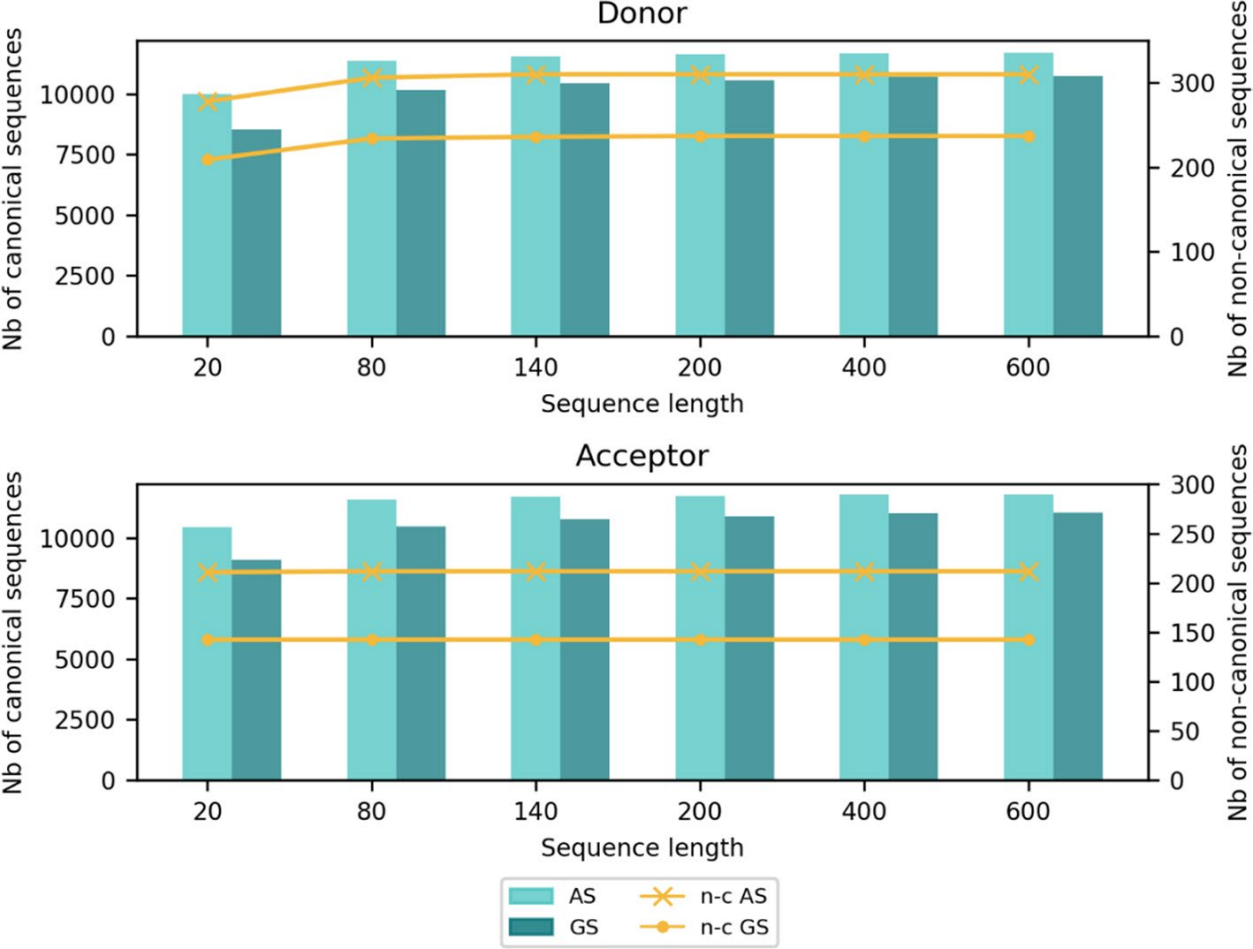
Number of canonical (bar) and non-canonical (n-c) (line) sequences for each positive subset (AS and GS) and for each sequence length

**Fig. 10:**
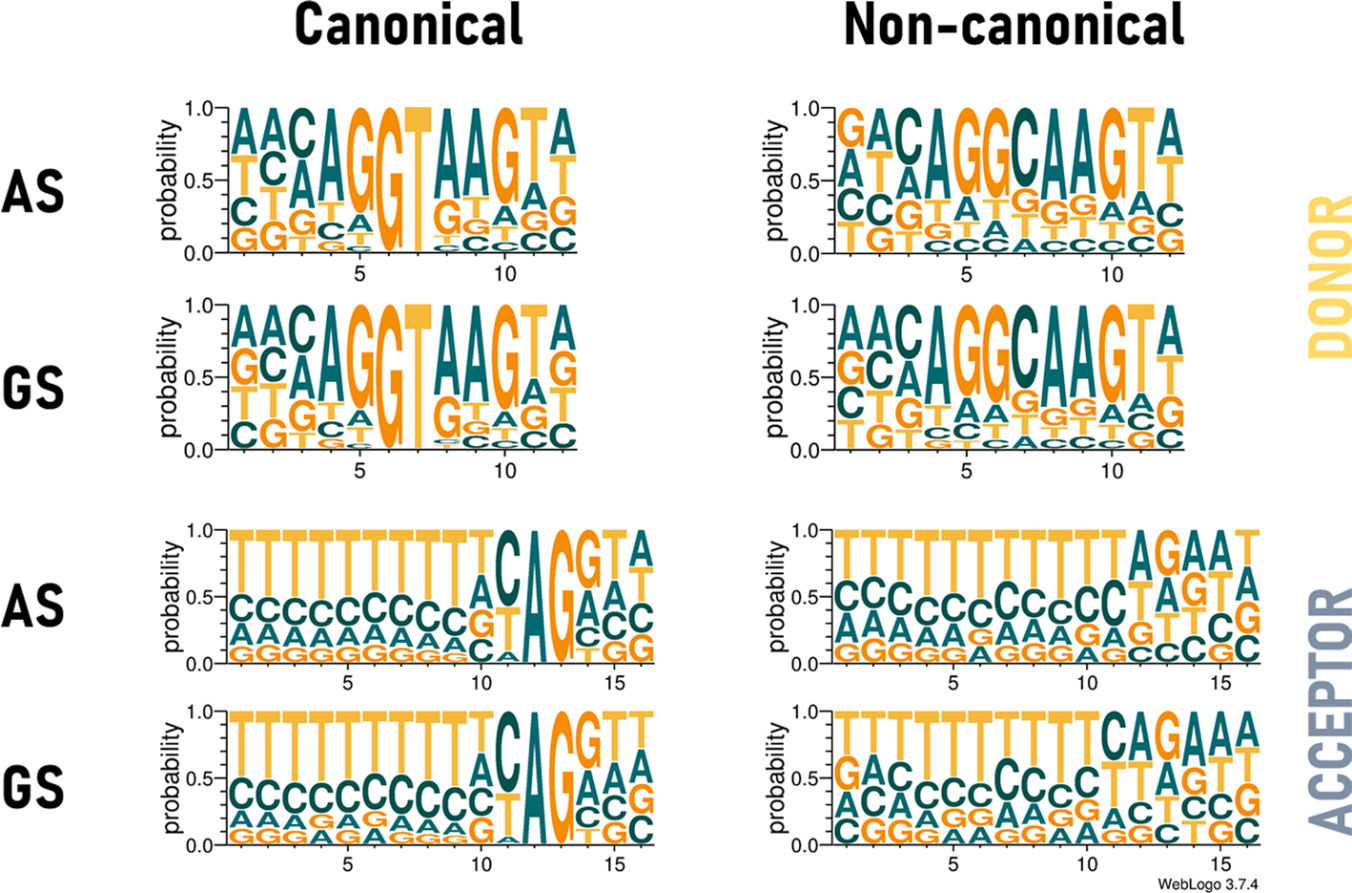
Sequence logos for canonical and non-canonical SS for each SS type (donor or acceptor) and each positive subset (AS and GS)

In order to test the impact of the genomic context, each dataset is provided in six different versions, according to the defined length of the input sequences. Sequence lengths selected for this study are 20, 80, 140, 200, 400 and 600 nt, where the SS is always in the central position. To reduce redundancy, for each sequence length, duplicate sequences are removed. As the length of the sequences decreases, the number of duplicates increases, reducing the size of the data sets (especially for 20 nt sequences). Figure 9 (Additional file 1: Table S5) summarizes the composition of the AS and GS positive subsets according to sequence length. Each SS is described according to its type, either canonical (i.e. GT for donor site and AG for acceptor site) or non-canonical. The number of non-canonical donor and acceptor SS present in each AS and GS dataset for each sequence length is also shown in Fig. 9 (Additional file 1: Table S5). In addition, Fig. 10 shows the sequence logos of the canonical and non-canonical donor and acceptor SS motifs from the AS and GS dataset sequences. The sequence logos were made with the program WebLogo v3.7.4 [75].

### Negative subsets

Two negative subsets were first constructed. The first one is composed only of FP sequences, i.e. randomly selected regions in the G3PO+ sequences in both 'Confirmed' and ‘Unconfirmed’ sequences (for AS dataset) or only ‘Confirmed’ sequences (for GS dataset), with a GT or AG dinucleotide (depending on the type of SS), in the central position (e.g. 301–302 for length = 600 nt) that do not correspond to a SS identified in the positive subsets. The second type of negative subset is composed of 3 categories of sequences extracted from the G3PO+ dataset:

- Exon sequences: randomly selected exon regions,
- Intron sequences: randomly selected intron regions,
- False positive SS: randomly selected GT or AG dinucleotides.

Negative subsets were also constructed with different numbers of sequences depending on the size of the positive subset: (i) with a ratio 1:1 we have the same number of positive and negative sequences, (ii) with a ratio 1:2 we have twice as many negative sequences as positive sequences and (iii) with a ratio 1:10 we have ten times more negative sequences than positive sequences. As for the positive subsets, identical redundant sequences and sequences containing undetermined ‘N’ characters were removed. Finally, as the selection of the negative sequences is random, 10 random selections were made, in order to obtain 10 different negative subsets and to eliminate potential random bias due to a specific data sampling.

### Data composition strategies

By combining the same positive subset with different negative subsets, a number of datasets were constructed in order to measure the impact of different parameters, including the type of negative examples used (heterogeneous = exons, introns and FP or homogeneous = only FP), the use of balanced or unbalanced datasets defined by the ratio of positive to negative examples, and data quality (AS vs. GS). In total, eight datasets were established, summarized in Table 4.

**Table 4:** Composition of the 8 datasets

Dataset	Quality of sequences	No. of positive sequences			No. of negative sequences	Type of negative sequences	Ratio
		Donor		Acceptor			
AS_0	Unconfirmed and confirmed	12,000	12,000		12,000	FP only	1:1
AS_1					12,000	4000 exons, 4000 introns and 4000 FP	
AS_2					24,000	FP only	1:2
AS_10					120,000	FP only	1:10
GS_0	Confirmed	10,973	11,179		11,000	FP only	1:1
GS_1					11,000	3650 exons, 3650 introns and 3700 FP	
GS**_**2					22,000	FP only	1:2
GS**_**10					110,000	FP only	1:10

Composition of the 8 datasets used to study the impact of (i) the type of negative examples (only FP sequences vs. heterogeneous data with exons, introns and FP sequences), (ii) the ratio of positive to negative examples (1:1, 1:2 and 1:10), (iii) data quality (‘Confirmed’ and ‘Unconfirmed’ sequences in the AS datasets vs. only Confirmed sequences in the GS datasets

FP, False Positive; GS, Gold Standard; AS, All Sequences

### Sequence identity

A sequence similarity search was performed on the whole sequences with a length of 600 nt and 20 nt from the AS and GS positive subsets. Each sequence was compared to all the others and the pairwise percent identity was defined by:

$$\% Identity = \left( {\frac{Number\,of\,identical\, nucleotide}{{Length\,of\,sequence}}} \right)*100$$

### Preparation of datasets for CNN

#### Training and test sets

**Fig. 11:**
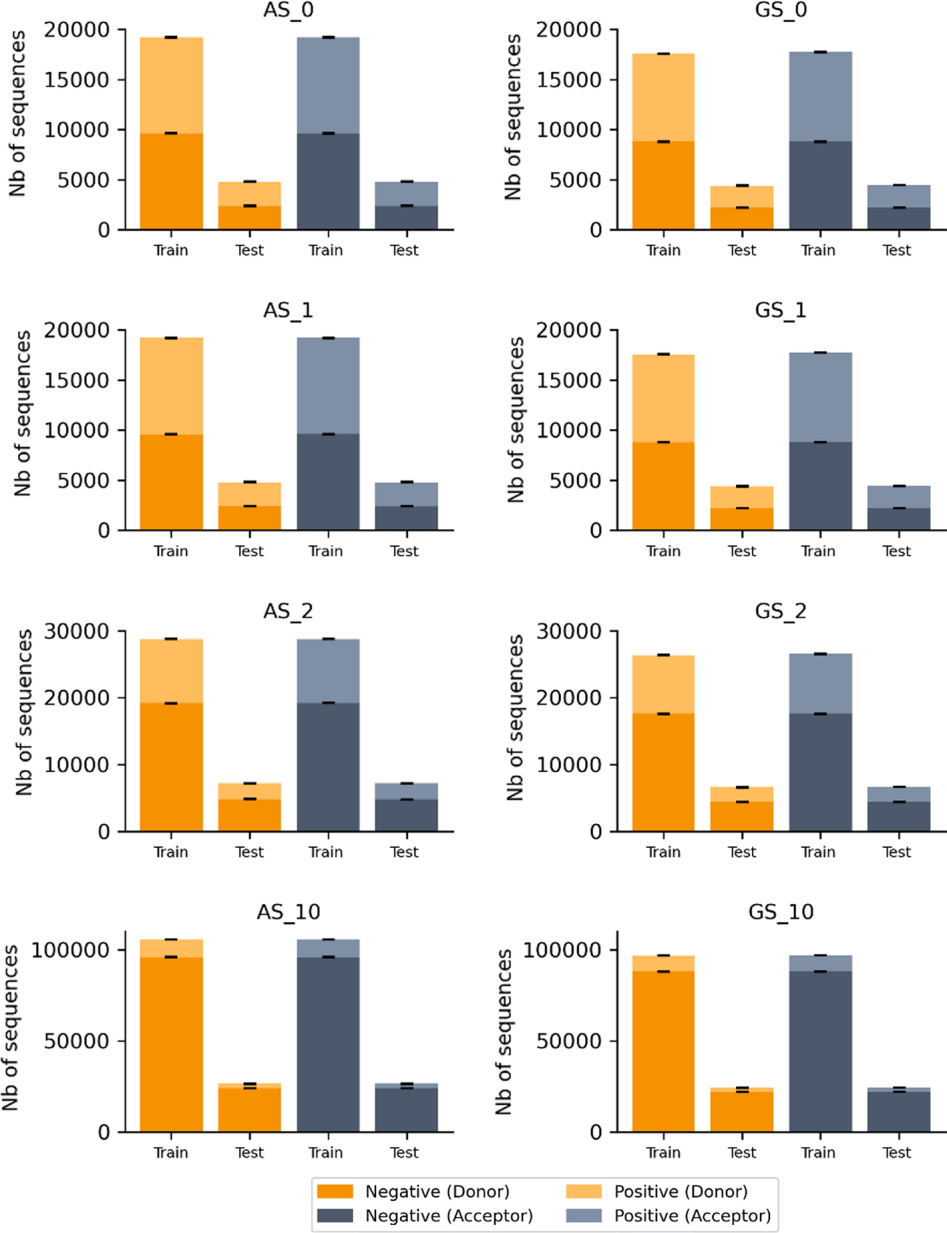
Average number of positive and negative sequences in training and test sets, for all AS and GS datasets, according to SS type. Standard deviations are indicated by black bars

For each dataset described above, a random selection was performed to form a training set containing 80% of the sequences and a test set containing 20% of the sequences. Since there are 10 different negative datasets, there are 10 different training and test sets. Figure 11 (Additional file 1: Table S6 A and B) summarizes the average number of negative and positive sequences in all training and test sets for each dataset (AS and GS) and for donor and acceptor SS.

#### Data encoding

**Fig. 12:**
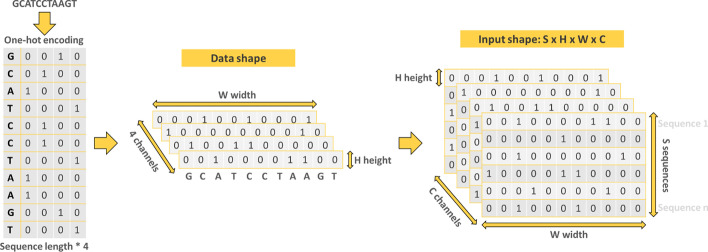
Data pre-processing. Input sequences are converted in one-hot encoding. The result is a 1D vector of size W, where W is the length of the input sequences, with 4 channels

For efficient exploitation of the genomic sequences in the training and test sets, a one-hot encoding step was performed. Each nucleotide of an input sequence was converted into a binary vector of size 4. Adenine is encoded by the vector (1,0,0,0), Cytosine by (0,1,0,0), Guanine by (0,0,1,0) and finally Thymine is encoded by (0,0,0,1). In the case where an external sequence contains indeterminate ‘N’ nucleotides, *e.g*. when users test their own sequences, the vector (0,0,0,0) is used. Thus, each output sequence is a first order tensor (vector) of size W, where W is the length of the input sequence, with 4 channels representing the one-encoding. Finally, the shape of the input is: S × H × W × C, where S is the number of input sequences, H is the height of the 1D vector (so H = 1), W is the width of the vector corresponding to the length of the input sequences and C is the number of channels from one-hot encoding. Figure 12 summarizes the data encoding.

Finally, the shape of the input is S × H × W × C (S = number of input sequences, H = height of the vector (here equal to 1 because the vector is 1 dimensional), W is the length of each input sequence and C is the number of channels).

### Convolutional Neural Network

Models were constructed and trained for each type of SS (donor or acceptor) independently. The models result from supervised learning, where genomic sequences (input) are coupled with class labels 0: non-SS and 1: SS. The CNN then applies filters on each input sequence and tries to modify the weights of these filters to improve the predictions thanks to the back-propagation algorithm. The filters allow to extract pertinent features/patterns within the input data. The output of the CNN is a vector of size 2, corresponding to the non-SS (0)/SS classes (1). The implementation of the CNN, as well as the training of the models, was done in Python v3.7, with Tensorflow v2.4.1 [76], the API Keras v2.3.1 and the Scikit-learn library v0.23.2 [77].

#### Architecture

**Fig. 13:**
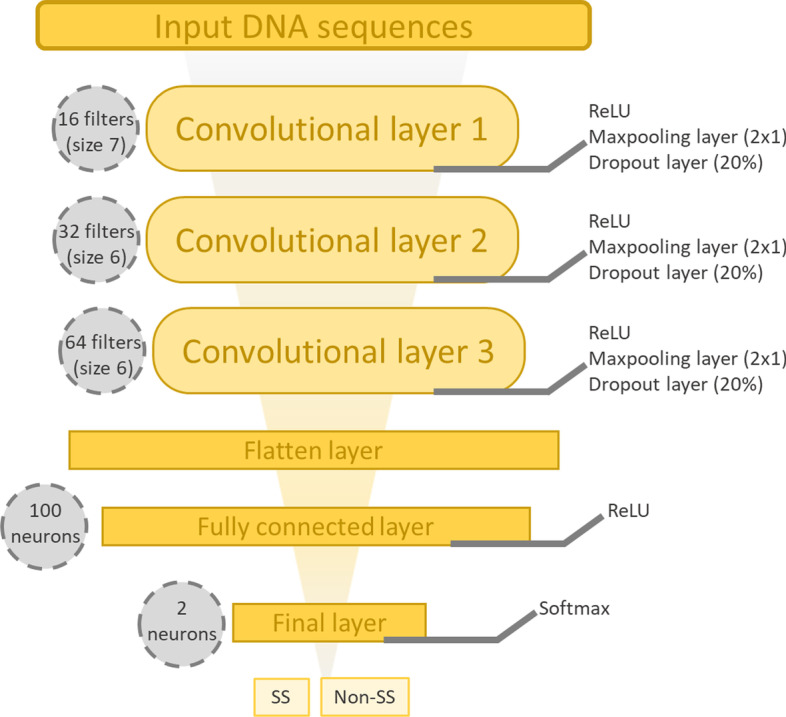
Representation of the CNN architecture. The architecture is composed of 2 convolutional layers, each followed by a dropout step and maxpooling layer. Then, a flatten layer is added to flatten the input. The output layer consists of 2 neurons activated by the Softmax function

We constructed a CNN architecture for donor or acceptor prediction, composed of a series of three convolutional layers over a single spatial dimension. The layers are composed of 16, 32 and 64 filters of sizes 7, 6 and 6 respectively, with a stride of 1. Between each convolution layer, a maxpooling layer of size 2 × 1 with a stride of 2 is added. Maxpooling allows to under-sample the data by reducing their size, while preserving the features that seem important. A dropout layer [78] is also added between each convolution layer to inactivate 20% of neurons. Then, a data flattening step (flatten layer) is performed in order to generate a vector exploitable by the fully connected layer containing 100 neurons. The last layer is the final output layer, containing two neurons that return the results of the classification. The neurons of each layer are activated by a Rectified Linear Unit (ReLU) activation function, except for the last layer where the activation function is Softmax in order to establish probabilities for each neuron and thus to predict a class according to the highest probability. Figure 13 summarizes the CNN architecture.

#### Training process

During the training process, the training set is divided into two parts, to generate an evaluation set (containing 15% of the sequences) that allows to control the learning of the network and avoid overfitting. The cross-entropy function is used as a loss function and the Adamax optimization algorithm [79] is applied with a learning-rate of 1e^−5^. Finally, the training is performed during 400 epochs with a batch-size of 32.

### Evaluation

#### Metrics

SS are considered as true positives (TP) if they are correctly predicted and false positives (FP) otherwise. Nucleotides that do not correspond to a SS are considered as True Negatives (TN) if they are not predicted to be SS and False Negatives (FN) otherwise. To evaluate the performance of the CNN models, five metrics were used:

Accuracy is the ratio of the number of correct predictions to the total number of predictions:

$$Accuracy = \frac{{\left( {TP + TN} \right)}}{{\left( {TP + TN + FP + FN} \right)}}$$

Precision is the ratio of the number of correctly predicted SS to the total number of predicted SS:

$$Precision = \frac{TP}{{\left( {TP + FP} \right)}}$$

Sensitivity (also known as recall) is the ratio of the number of correctly predicted SS to the total number of SS:

$$Sensitivity = \frac{TP}{{\left( {TP + FN} \right)}}$$

Specificity is the ratio of the number of correctly predicted non-SS sequences to the total number of non-SS sequences:

$$Specificity = \frac{TN}{{\left( {TN + FP} \right)}}$$

F1 Score is the harmonic mean of the precision and sensitivity and shows a balance between these two metrics:

$$F1\, score = 2*\frac{Precision*Sensitivity}{{Precision + Sensitivity}}$$

#### Independent benchmarks of SS from model and non-model organisms

To estimate the reliability and robustness of the CNN models, they were evaluated on 5 large-scale benchmarks, including sequences from: Human, *D. rerio* (Fish), *D. melanogaster* (Fly), *C. elegans* (Worm) and *A. thaliana* (Plant), that were downloaded from https://public.bmi.inf.ethz.ch/user/behr/splicing/ [31]. A selection of 10,000 SS and 10,000 non-SS sequences was performed for each benchmark, including a number of non-canonical SS (human: 307; fish: 85; fly:120; worm: 67 and plant: 122).

To evaluate the performance of the models on non-model organisms, we constructed one other independent benchmark called ‘PVP’ (Protist and ViridiPlantae), containing sequences from protists and viridiplantae. The sequence selection process is similar to that used in G3PO. The reference sequences are the cytoplasmic tryptophanyl-tRNA synthetase of *Paramecium tetraurelia* (A0D783_PARTE) and the tryptophan-tRNA ligase of *Arabidopsis thaliana* (SYWM_ARATH). All orthologs were extracted from the OrthoInspector database version 3 [74], multiple sequence alignments were obtained with PipeAlign version 2 [80], and manually analyzed to identify 62 ‘Confirmed’ sequences (33 plants and 29 protists). Finally, the benchmark contains 692 (with 21 non-canonical) donor SS and 714 (with 18 non-canonical) acceptor SS, and the same number of non-SS sequences to balance the dataset.

The benchmarks were used to compare Spliceator with other existing SS prediction methods, including NNSplice, MaxEntScan, DSSP and SpliceFinder. Note that SpliceRover was not included in these large-scale benchmark tests, since the method is only available as a web server. Moreover, for a fair evaluation, only tools using the raw DNA sequences as features were included. Hence, SpliceAI was also not selected because the input file must be in Variant Call Format (.vcf) and not a raw sequence. In addition, SpliceAI models were trained with GENCODE data coupled with experimental data.

#### Explicability

The visualization heatmaps of the nucleotides most used by the models were generated using the Grad-CAM (Gradient Class Activation Map) technique [81]. The maps were generated from the training sets: the higher the score, the warmer the color (yellow) and the lower the score, the colder the color (deep blue). In order to highlight the most representative patterns identified during the training process, the heatmaps were averaged from 10,000 samples for each class of each SS.

## Supplementary Information

**Additional file 1.** Figures S1–S2 and Tables S1–S6

**Additional file 2.** Tables S1–S3, performance results of donor and acceptor models

## Data Availability

Spliceator source code (python) and the datasets analyzed or generated are available at: https://git.unistra.fr/nscalzitti/spliceator, and a web service is available at: www.lbgi.fr/spliceator/.

## Abbreviations

NGS: Next generation sequencing
RNA-seq: MRNA-sequencing
UTR: UnTranslated Region
SS: Splice Site
ML: Machine Learning
DL: Deep Learning
CNN: Convolutional Neural Network
BPS: BranchPoint site
ISE: Intronic Splicing Enhancer
ISS: Intronic Splicing Silencer
ESE: Exonic Splicing Enhancer
ESS: Exonic Splicing Silencer
FP: False Positive
AS: All Sequences
GS: Gold Standard
nt: Nucleotide
PPT: PolyPyrimidine Tract
PVP: Protists and ViridiPlantea
BBS: Bardet-Biedl Syndrome
ReLU: Rectified Linear Unit
TP: True Positive
FP: False Positive
TN: True Negative
FN: False Negative
