# Supplementary material for: Spliceator: multi-species splice site prediction using convolutional neural networks
Source: BMC Bioinformatics. 2021 Nov 23;22:561. doi: 10.1186/s12859-021-04471-3 (PMC8609763; doi:10.1186/s12859-021-04471-3)
Supplement: Supplementary file 1 — Additional file 1. Figures S1–S2 and Tables S1–S6 [file 12859_2021_4471_MOESM1_ESM.pdf]

			1	2	3	4	5	6	7	8	9	10	Avg.	Std. dev
Donor	AS	0	92.62	92.33	93.35	93.71	93.33	92.85	92.81	92.60	93.67	93.59	93.09	0.50
		1	93.44	93.02	93.73	93.98	94.25	93.92	94.32	94.00	94.40	94.46	93.95	0.46
		2	94.20	92.92	94.27	93.75	94.18	94.96	93.57	93.35	94.15	93.43	93.88	0.59
		10	97.33	97.45	97.41	97.65	96.99	97.33	97.36	97.10	97.27	97.37	97.33	0.18
	GS	0	93.44	94.03	93.80	93.90	94.63	94.31	94.28	94.36	94.59	93.78	94.11	0.39
		1	95.71	95.59	95.09	95.53	95.18	95.02	95.55	95.36	95.48	94.86	95.34	0.28
		2	95.22	95.53	94.86	95.70	94.66	94.80	94.71	95.29	95.15	95.19	95.11	0.35
		10	97.79	97.85	97.87	97.71	97.53	97.79	97.62	97.77	97.30	97.57	97.68	0.18
Acceptor	AS	0	92.21	93.05	92.82	91.25	92.21	91.86	92.13	91.00	92.09	91.31	91.99	0.66
		1	93.22	92.86	91.61	93.40	92.07	92.36	92.88	92.69	92.00	93.15	92.62	0.59
		2	94.35	93.95	93.55	93.28	92.85	94.01	93.95	93.74	92.73	94.58	93.70	0.60
		10	97.17	97.48	97.23	97.06	97.07	97.23	97.17	97.33	96.92	97.08	97.17	0.16
	GS	0	93.53	92.78	92.19	93.01	92.59	93.34	93.37	92.62	91.48	92.41	92.73	0.62
		1	94.41	94.00	94.46	94.25	94.41	94.00	94.23	94.21	94.12	93.80	94.19	0.21
		2	94.47	93.14	94.79	94.50	94.49	93.59	94.73	94.10	95.11	93.96	94.29	0.60
		10	97.26	97.38	97.54	97.59	97.60	97.35	97.47	97.54	97.21	97.53	97.45	0.14

Table S1 – Prediction accuracy for donor and acceptor SS, using the AS and GS datasets (AS/GS_0 = positive/negative ratio of 1:1 with only FP sequences in negative subset; AS/GS_1 = positive/negative ratio of 1:1 with exon, intron and FP sequences; AS/GS_2 = positive/negative ratio of 1:2 with only FP sequences in negative subset; AS/GS_10 = positive/negative ratio of 1:10 with only FP sequences in negative subset), with an input sequence length of 200 nt.

SS	Dataset comparison	P-value	Significant
Donor	AS_0 vs GS_0	1.23×10^{-5}	***
	AS_1 vs GS_1	5.88×10^{-9}	***
	AS_2 vs GS_2	3.7×10^{-6}	***
	AS_10 vs GS_10	9.13×10^{-5}	***
Acceptor	AS_0 vs GS_0	1.0×10^{-2}	***
	AS_1 vs GS_1	3.09×10^{-8}	***
	AS_2 vs GS_2	2.4×10^{-2}	**
	AS_10 vs GS_10	1.87×10^{-4}	***

Table S2 - Statistical analysis with unpaired t-tests for difference between average prediction accuracy for donor and acceptor SS using AS and GS datasets (from Table S1) (**: p-value < 0.01 and ***: p-value < 0.001).

A) Donor

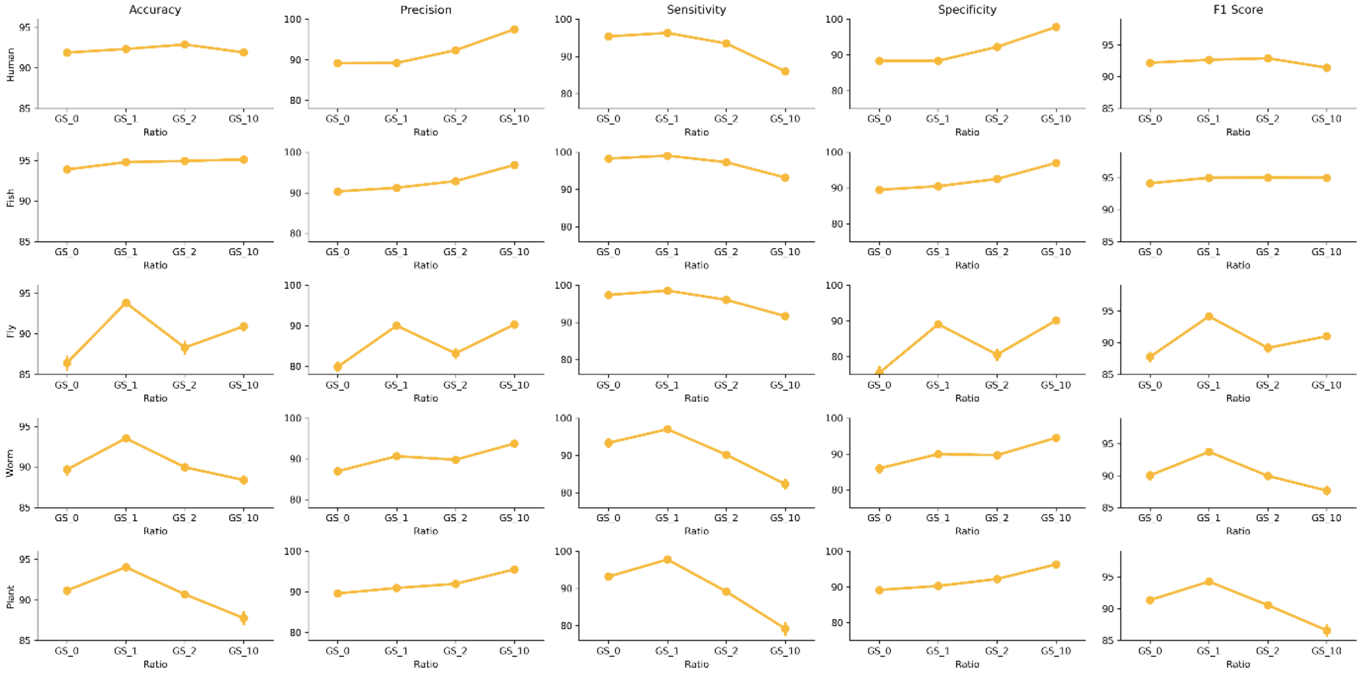

B) Acceptor

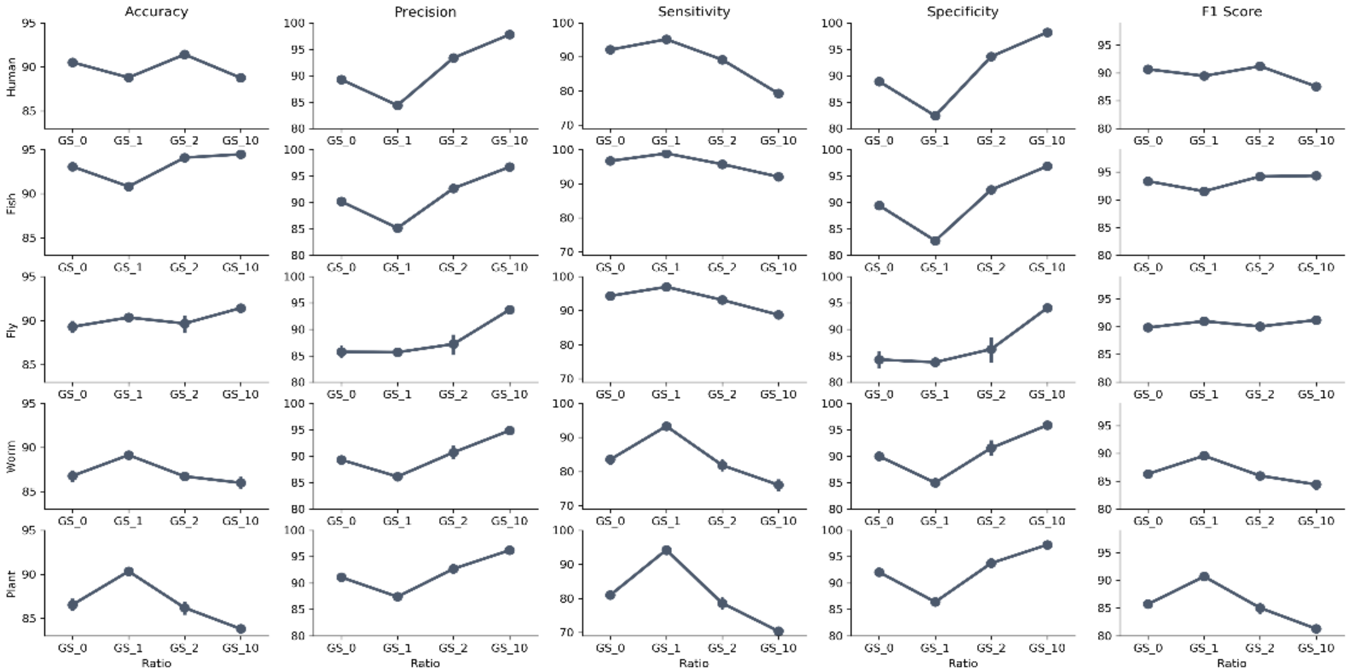

Figure S1 – Average performance for GS datasets and for A) donor and B) acceptor SS, on 5 different benchmarks (human, fish, fly, worm, plant). Five metrics are used to evaluate the performance: accuracy, precision, sensibility, specificity and F1 score. AS/GS_0 = positive/negative ratio of 1:1 with only FP sequences in negative subset; AS/GS_1 = positive/negative ratio of 1:1 with exon, intron and FP sequences; AS/GS_2 = positive/negative

ratio of 1:2 with only FP sequences in negative subset; AS/GS_10 = positive/negative ratio of 1:10 with only FP sequences in negative subset.

		1	2	3	4	5	6	7	8	9	10	Avg	Std. Dev
Donor	Accuracy	95.71	95.59	95.09	95.53	95.18	95.02	95.55	95.36	95.48	94.86	95.34	0.28
	Precision	93.40	92.87	92.88	91.69	92.67	92.49	92.60	91.39	93.59	91.39	92.50	0.78
	Sensitivity	98.50	98.48	98.56	98.38	98.12	98.21	98.22	98.36	98.68	97.60	98.31	0.30
	Specificity	92.91	92.94	92.68	91.20	92.25	92.00	92.06	91.08	93.08	90.93	92.11	0.81
	F1 score	95.88	95.59	95.64	94.92	95.32	95.27	95.33	94.75	96.07	94.39	95.32	0.52
Acceptor	Accuracy	94.41	94.00	94.46	94.25	94.41	94.00	94.23	94.21	94.12	93.80	94.19	0.21
	Precision	91.77	91.49	91.58	91.64	92.58	91.52	92.36	91.42	91.51	91.45	91.73	0.40
	Sensitivity	97.87	97.17	97.80	97.30	96.73	97.00	96.77	97.76	97.04	96.58	97.20	0.47
	Specificity	90.78	90.77	91.18	91.25	92.04	91.00	91.55	90.54	91.28	91.04	91.14	0.43
	F1 score	94.72	94.25	94.59	94.38	94.61	94.18	94.51	94.49	94.20	93.95	94.39	0.24

Table S3 - Optimized Spliceator model performance averaged over 10 experiments. Optimal model corresponds to the GS_1 dataset, with input sequence length of 200 nt, high quality positive sequences, a balanced number of positive and negative sequences and heterogeneous negative examples (exon, intron and FP sequences).

Gene Name	Gene length (nt)	No. of orthologous sequences	Gene Name	Gene length (nt)	No. of orthologous sequences
ARL6	**36709**	**119**	*LMOD3*	16161	43
BBIP1	**20545**	**36**	***LZTL1***	**92727**	**79**
BBS1	**23008**	**127**	*MEG10*	133936	41
BBS10	**3969**	**37**	***MKKS***	**33214**	**54**
BBS12	**12241**	**44**	***MKS1***	**14169**	**90**
BBS2	**53252**	**119**	*MTM1*	104704	39
BBS4	**52297**	**130**	*MY18B*	288898	37
BBS5	**27176**	**125**	*MYPC3*	21306	42
BBS7	**46058**	**117**	*MYPN*	105896	44
BIN1	59329	40	*NEBU*	247397	43
CELN	18029	39	***PTHB1***	**476825**	**131**
CH037	**24283**	**66**	*RASH*	5046	36
CNTN1	379977	41	*ROA1*	6896	30
COF2	8055	47	*RYR1*	153591	27
DYN2	115410	42	***SDCG8***	**244037**	**54**
FRITZ	**467417**	**71**	*SPEG*	63442	45
HACD1	28346	36	*SPTN1*	110224	32
HUWE1	122012	31	*TMM8C*	10361	38
IFT27	**18055**	**85**	*TPM2*	9029	14
IFT74	**115892**	**131**	*TPM3*	39345	24
KBTBD	1377	45	***TRI32***	**13999**	**40**
KLH40	7026	50	***TTC8***	**56926**	**138**
KLH41	16561	42			

Table S4 – 45 Human reference genes used in the G3PO+ dataset (original G3PO genes are in bold and the others genes correspond to the extended set G3PO+)

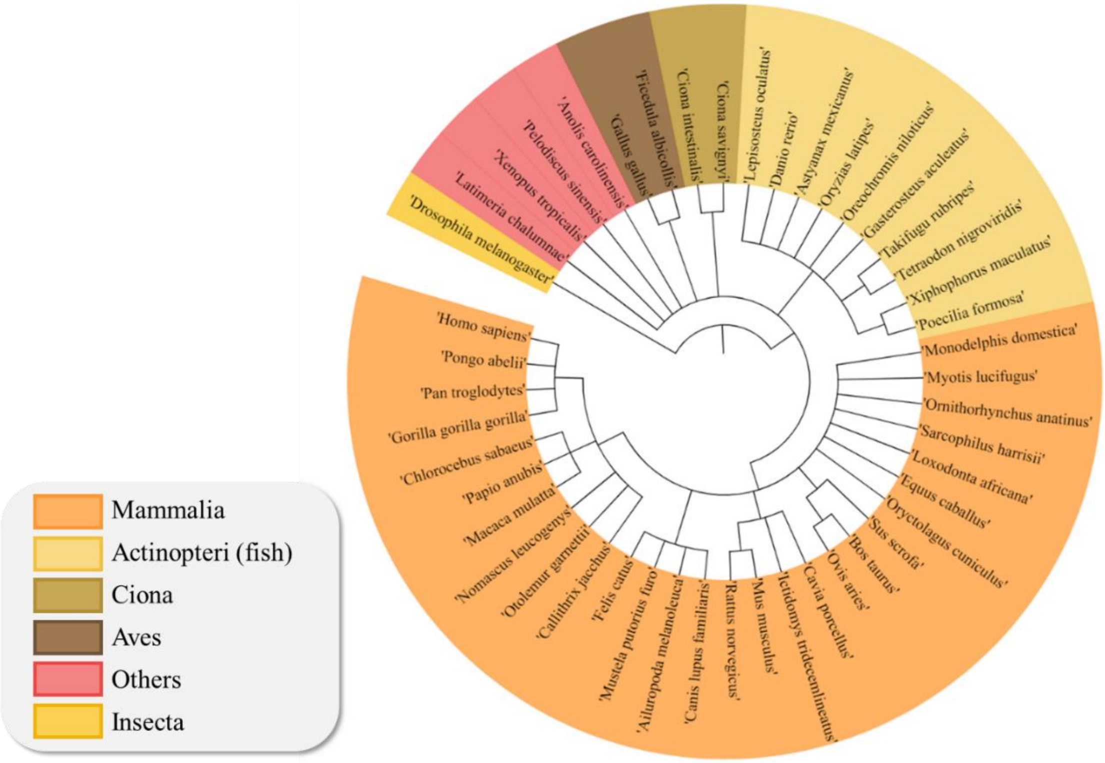

Figure S2 – List of metazoans added in G3PO+ (Phylogenetic tree built with iTOL v5) (Letunic and Bork, 2021).

Length	**Canonical**			
	Donor		Acceptor	
	AS	GS	AS	GS
20	9982	8519	10440	9091
80	11348	10159	11564	10463
140	11551	10440	11694	10751
200	11619	10553	11736	10873
400	11675	10708	11780	11013
600	12000	10736	11788	11036
**Non-canonical**				
20	278 (2.71%)	209 (2.39%)	211 (1.98%)	143 (1.55%)
80	306 (2.63%)	234 (2.25%)	212 (1.80%)	143 (1.35%)
140	310 (2.61%)	236 (2.21%)	212 (1.78%)	143 (1.31%)
200	310 (2.60%)	237 (2.20%)	212 (1.77%)	143 (1.30%)
400	310 (2.59%	237 (2.17%)	212 (1.77%)	143 (1.28%)
600	310 (2.52%)	237 (2.16%)	212 (1.77%)	143 (1.28%)
**Total**				
20	10260	8728	10651	9234
80	11654	10393	11776	10606
140	11861	10676	11906	10894
200	11929	10790	11948	11016
400	11985	10945	11992	11156
600	12310	10973	12000	11179

Table S5 – Number of canonical and non-canonical sequences for AS and GS positive datasets for each input sequence length and for each SS (donor and acceptor).

[A] DONOR		Set	1	2	3	4	5	6	7	8	9	10	Avg.
AS_0	Negative sequences	Train	9649	9629	9596	9578	9583	9673	9636	9553	9649	9639	9619
		Test	2350	2370	2403	2421	2416	2326	2363	2446	2350	2360	2381
	Positive sequences	Train	9550	9570	9603	9621	9616	9526	9563	9646	9550	9560	9581
		Test	2450	2430	2397	2379	2384	2474	2437	2354	2450	2440	2420
AS_1	Negative sequences	Train	9562	9555	9592	9581	9572	9634	9599	9605	9582	9590	9587
		Test	2438	2445	2408	2419	2428	2366	2401	2395	2418	2410	2413
	Positive sequences	Train	9635	9642	9607	9618	9626	9566	9601	9594	9617	9609	9612
		Test	2362	2355	2392	2381	2372	2434	2399	2405	2382	2390	2387
AS_2	Negative sequences	Train	19203	19130	19139	19157	19147	19225	19169	19170	19192	19175	19171
		Test	4794	4868	4858	4840	4850	4772	4829	4828	4805	4822	4827
	Positive sequences	Train	9594	9668	9658	9640	9650	9572	9629	9628	9605	9622	9627
		Test	2406	2332	2342	2360	2350	2428	2371	2372	2395	2378	2373
AS_10	Negative sequences	Train	95992	95956	95942	95986	96081	95959	95908	95946	95950	95984	95970
		Test	23985	24019	24031	23993	23893	24016	24066	24032	24021	23989	24005
	Positive sequences	Train	9589	9624	9636	9597	9498	9621	9671	9636	9626	9594	9609
		Test	2411	2376	2364	2403	2502	2379	2329	2364	2374	2406	2391
GS_0	Negative sequences	Train	8799	8831	8760	8734	8855	8817	8803	8795	8834	8820	8805
		Test	2201	2169	2240	2266	2145	2183	2197	2205	2166	2180	2195
	Positive sequences	Train	8779	8747	8818	8844	8723	8761	8775	8783	8744	8758	8773
		Test	2194	2226	2155	2129	2250	2212	2198	2190	2229	2215	2200
GS_1	Negative sequences	Train	8746	8779	8755	8798	8759	8823	8808	8775	8819	8754	8782
		Test	2254	2221	2245	2202	2241	2177	2192	2225	2181	2246	2218
	Positive sequences	Train	8831	8797	8823	8780	8818	8755	8770	8801	8757	8822	8795
		Test	2141	2174	2150	2193	2154	2218	2203	2169	2213	2149	2176
GS_2	Negative sequences	Train	17548	17600	17550	17580	17587	17668	17585	17576	17613	17559	17587
		Test	4452	4400	4450	4420	4413	4332	4415	4424	4387	4441	4413
	Positive sequences	Train	8830	8778	8828	8798	8791	8710	8793	8802	8765	8819	8791
		Test	2143	2195	2145	2175	2182	2263	2180	2171	2208	2154	2182
GS_10	Negative sequences	Train	88064	87974	88048	87986	88037	87965	88019	87890	88099	88023	88011
		Test	21936	22026	21952	22014	21963	22035	21981	22110	21901	21977	21990
	Positive sequences	Train	8714	8804	8730	8792	8741	8813	8759	8888	8679	8755	8768
		Test	2259	2169	2243	2181	2232	2160	2214	2085	2294	2218	2206

Table S6A – Number of positive and negative sequences in the 10 training and test sets for each AS and GS dataset and for donor SS.

[B] ACCEPTOR		Set	1	2	3	4	5	6	7	8	9	10	Avg.
AS_0	Negative sequences	Train	9536	9614	9627	9598	9601	9596	9616	9634	9648	9584	9605
		Test	2462	2385	2371	2401	2398	2403	2383	2365	2351	2415	2393
	Positive sequences	Train	9662	9585	9571	9601	9598	9603	9583	9565	9551	9615	9593
		Test	2338	2415	2429	2399	2402	2397	2417	2435	2449	2385	2407
AS_1	Negative sequences	Train	9627	9558	9619	9577	9593	9605	9613	9630	9600	9628	9605
		Test	2373	2442	2381	2423	2407	2395	2387	2370	2400	2372	2395

	Positive sequences	Train	9573	9640	9580	9623	9607	9595	9587	9569	9600	9571	9595
		Test	2427	2358	2419	2377	2393	2405	2413	2430	2400	2428	2405
AS_2	Negative sequences	Train	19201	19231	19189	19204	19206	19169	19227	19231	19265	19231	19215
		Test	4794	4765	4807	4793	4789	4829	4771	4767	4733	4767	4782
	Positive sequences	Train	9595	9565	9607	9593	9590	9629	9571	9567	9533	9567	9582
		Test	2405	2435	2393	2407	2410	2371	2429	2433	2467	2433	2418
AS_10	Negative sequences	Train	95966	96014	95909	95972	95924	96066	96019	96018	95986	95993	95987
		Test	24012	23970	24072	24012	24052	23914	23959	23966	23996	23989	23994
	Positive sequences	Train	9616	9573	9675	9615	9656	9518	9563	9569	9599	9592	9598
		Test	2384	2427	2325	2385	2344	2482	2437	2431	2401	2408	2402
GS_0	Negative sequences	Train	8812	8776	8775	8771	8744	8799	8830	8791	8847	8806	8795
		Test	2188	2224	2225	2229	2256	2201	2170	2209	2153	2194	2205
	Positive sequences	Train	8931	8967	8968	8972	8999	8944	8913	8952	8896	8937	8948
		Test	2248	2212	2211	2207	2180	2235	2266	2227	2283	2242	2231
GS_1	Negative sequences	Train	8820	8832	8782	8761	8830	8802	8815	8780	8828	8846	8810
		Test	2180	2168	2218	2239	2170	2198	2185	2220	2172	2154	2190
	Positive sequences	Train	8923	8910	8960	8981	8912	8941	8927	8961	8915	8896	8933
		Test	2256	2268	2218	2197	2266	2238	2251	2216	2264	2282	2246
GS_2	Negative sequences	Train	17590	17580	17591	17626	17578	17520	17612	17579	17619	17629	17592
		Test	4410	4420	4409	4374	4422	4480	4388	4421	4381	4371	4408
	Positive sequences	Train	8953	8963	8952	8917	8965	9023	8931	8964	8924	8914	8951
		Test	2226	2216	2227	2262	2214	2156	2248	2215	2255	2265	2228
GS_10	Negative sequences	Train	87973	88023	87923	87921	87967	88008	88013	88009	88048	88008	87989
		Test	22027	21977	22077	22079	22033	21992	21987	21991	21952	21992	22011
	Positive sequences	Train	8970	8920	9020	9022	8976	8935	8930	8934	8895	8935	8954
		Test	2209	2259	2159	2157	2203	2244	2249	2245	2284	2244	2225

Table S6B – Number of positive and negative sequences in the 10 training and test sets for each AS and GS dataset and for acceptor SS.

References

Letunic,I. and Bork,P. (2021) Interactive Tree Of Life (iTOL) v5: an online tool for phylogenetic tree display and annotation. *Nucleic Acids Research*.
